# Keratin 19 as a prognostic marker and contributing factor of metastasis and chemoresistance in high‐grade serous ovarian cancer

**DOI:** 10.1002/1878-0261.70227

**Published:** 2026-02-19

**Authors:** Sophia Bielesch, Isabel Vogel, Sina Nokodian, Johanna Moeller, Antonia Blechschmidt, Vicky Hecht, Vanessa Kuentzel, Katharina Thiedig, Melissa Schwab, Oliver Schilling, Holger Bronger, Marion Kiechle, Viktor Magdolen, Tobias Dreyer

**Affiliations:** ^1^ Department of Gynaecology and Obstetrics Technical University of Munich Germany; ^2^ Faculty of Medicine, Institute for Surgical Pathology, Medical Centre University of Freiburg Germany; ^3^ German Cancer Consortium (DKTK) Partner Site Munich, a partnership between DKFZ and Technical University of Munich Heidelberg Germany

**Keywords:** chemoresistance, epithelial–mesenchymal plasticity, keratin 19, ovarian cancer

## Abstract

High‐grade serous ovarian cancer (HGSOC) is the most prevalent and lethal subtype of epithelial ovarian cancer (EOC), characterised by extensive peritoneal metastasis. The intermediate filament keratin 19 (KRT19) has been linked to tumour progression and chemoresistance in various cancers. However, its role varies across tumour types and remains unclear for HGSOC. We evaluated KRT19 protein expression in 199 HGSOC patients and correlated findings with clinical outcomes. *In vitro*, we assessed the effects of KRT19 on tumour‐associated mechanisms, including proliferation, migration, adhesion, and spheroid formation. A xenograft mouse model was used to assess tumour burden *in vivo*. Publicly available datasets enabled *in silico* validation. KRT19 was significantly overexpressed in HGSOC, and high expression was associated with reduced overall survival. *In vivo*, KRT19‐overexpression increased peritoneal tumour burden. *In vitro* and *ex vivo*, KRT19 induced a hybrid epithelial phenotype through enhanced epithelial–mesenchymal plasticity (EMP), promoting adhesion, migration, and spheroid integrity, thereby potentially supporting metastatic processes. Further, KRT19 could contribute to paclitaxel resistance. Altogether, KRT19 represents a potential independent prognostic marker and therapeutic target to inhibit metastatic dissemination.

## Graphical abstract

Keratin 19 (KRT19) is overexpressed in high‐grade serous ovarian cancer with high levels of Kallikrein‐related peptidases (KLK) 4–7 and is associated with poor survival. *In vivo* analyses demonstrate that elevated KRT19 increases peritoneal tumour burden. Functionally, KRT19 promotes epithelial–mesenchymal plasticity, enhancing adhesion, migration, and spheroid integrity, thereby supporting metastatic dissemination and reducing overall survival.

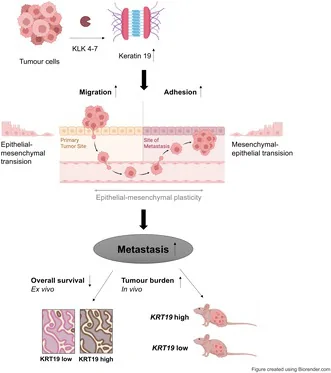

**Abbreviations**
ATCs: ascites tumour cells
CNV: copy number variations
Col IV: collagen IV
DAB: 3,3′‐Diaminobenzidine
DAPI: 4′,6‐diamidin‐2‐phenylindole
EMP: epithelial–mesenchymal plasticity
EMT: epithelial–mesenchymal transition
EOC: epithelial ovarian cancer
ES: enrichment score
FBS: fetal bovine serum
FFPE: formalin‐fixed paraffin‐embedded
FIGO: Fédération Internationale de Gynécologie et d'Obstétrique
FN: fibronectin
GEO: Gene Expression Omnibus
GSEA: gene set enrichment analysis
GTEX: genotype‐tissue expression
HGSOC: high‐grade serous ovarian carcinoma
HRP: horseradish peroxidase
IHC: immunohistochemical
KRT19: keratin 19
MET: mesenchymal‐epithelial transition
N‐cadherin: neural cadherin
NES: normalised enrichment score
OC: ovarian cancer
RIPA: radioimmunoprecipitation assay
RT‐qPCR: reverse transcription‐quantitative PCR
SEM: standard error of the mean
TARGET: therapeutically applicable research to generate effective treatments
TBS‐T: tris‐buffered saline with tween 20
TCGA: The Cancer Genome Atlas
VN: vitronectin

## 1 Introduction

Ovarian cancer (OC) is the eighth leading cause of cancer in women and represents the deadliest gynaecologic malignancy [1]. Among its various subtypes, epithelial ovarian cancer (EOC) accounts for approximately 90% of all cases, and high‐grade serous ovarian cancer (HGSOC) is the most prevalent histological subtype [2]. A major challenge is that most patients are diagnosed at advanced stages (Fédération Internationale de Gynécologie et d'Obstétrique (FIGO) III/IV), with five‐year survival around 40% [3]. While platinum‐based chemotherapy represents the standard treatment, relapse and resistance frequently emerge [4]. Moreover, 80–100% of patients with advanced HGSOC exhibit peritoneal metastases at diagnosis, and distant metastatic spread is common [2]. This extensive metastatic potential is a principal contributor to the high mortality associated with OC. With limited therapeutic options and unclear molecular pathways underlying migration and metastasis, identifying new therapeutic targets is urgently needed.

Cancer progression, metastasis and chemoresistance are driven by a complex interplay of molecular factors in the cytoskeleton. Keratins, which form the intermediate filaments in epithelial cells, are vital for cell stability and integrity [5]. Although they provide mechanical support, keratins also regulate a range of processes, including cell proliferation, adhesion and migration, by reorganising intracellular structures and influencing signalling pathways [6, 7]. Phosphorylation of keratins can modulate their structural organisation, subsequently influencing cellular polarity and motility [8]. This capacity to modulate key processes is of particular interest in OC, where tumour cells must repeatedly adapt to a dynamic microenvironment. One key process is the formation of spheroids—often found in ascites fluid—that facilitate survival and metastatic colonisation by shielding cells from detachment‐induced cell death (anoikis) and immune clearance [9]. The dissemination of cancer cells into the ascitic fluid or bloodstream is primarily driven by epithelial–mesenchymal transition (EMT), which increases tumour cells' migratory ability [10]. However, detached cancer cells often partially undergo the reverse process—the mesenchymal–epithelial transition (MET)—to retain certain epithelial characteristics while acquiring mesenchymal traits, creating an intermediate phenotype that can be even more aggressive [11]. The dynamic combination of these processes is summarised under the term ‘epithelial‐mesenchymal plasticity’ (EMP) [10].

Another emerging concept relevant to HGSOC dissemination is the ‘go or grow’ paradigm, in which tumour cells allocate metabolic resources between proliferation (‘grow’) and migration (‘go’). In environments that demand substantial migratory capacity, cells may transiently downregulate proliferation to meet the energy requirements of movement [12]. Understanding how tumour cells navigate these trade‐offs could inform targeted therapies that disrupt their metastatic behaviour.

Within this context, keratin 19 (KRT19)—the smallest member of the type I keratin family—has drawn increased attention [13, 14]. In previous studies, we found significantly elevated *KRT19* levels in HGSOC patients with upregulated kallikrein‐related peptidases, which are linked to poor survival [15]. Although KRT19 has been implicated in tumour‐suppressive functions in certain cancer types, recent reports indicate that its upregulation can promote tumour progression, metastasis and poor outcomes in entities such as lung squamous cell carcinoma and hepatocellular carcinoma [13, 16]. Despite these indications, the precise role of KRT19 in HGSOC remains underexplored. By focusing on a well‐characterised HGSOC cohort and combining clinical data with functional assays, this study aimed to evaluate whether KRT19 serves as a prognostic marker and to elucidate its influence on the balance between proliferation and migration, cell spheroid formation and EMT‐related processes. Moreover, we assessed the impact of KRT19 on chemoresistance, particularly to paclitaxel‐ and platinum‐based agents, given their status as first‐line therapies in advanced OC.

The results of the present study suggest that KRT19 may contribute to OC's major clinical challenges, including metastatic dissemination and therapy resistance. By clarifying its mechanistic roles and clinical relevance, our investigations not only provide valuable insights into HGSOC tumour biology but also open new avenues for precision‐targeted interventions to improve the prognosis of patients with this aggressive malignancy.

## 2 Materials and methods

### 2.1 Immunohistochemical staining

5 μm sections of formalin‐fixed paraffin‐embedded (FFPE) tissue microarrays were cut, mounted on slides, deparaffinised (xylene, ethanol series) and rehydrated. Antigen retrieval was performed in citrate buffer (pH 6.0). Endogenous peroxidase was blocked with 3% H_2_O_2_. Sections were incubated with an anti‐KRT19 antibody (MAB 10725; Abnova, Taipei City, Taiwan; 1 : 200) for one hour at room temperature, followed by horseradish peroxidase HRP polymer (Zytomed, Berlin, Germany) for 30 min. 3,3'‐Diaminobenzidin (DAB) (Zytomed, Berlin, Germany) was applied for 8 min, and slides were counterstained with haematoxylin. Tris‐buffered saline with Tween 20 (TBS‐T) washes were performed between all steps. We have used a semi‐quantitative digital analysis of the immunohistochemistry (IHC) score as described before [17].

### 2.2 Cell culture

Human HGSOC cell lines OV‐MZ‐6 (RRID: CVCL_4005) (obtained from Möbus et al. [18]) and Kuramochi (RRID: CVCL_1345) (obtained from ATCC, Manassas, VA, USA) were cultured at 37 °C in 5% CO_2_. OV‐MZ‐6 cells were cultured in DMEM (Gibco, Thermo Fisher Scientific, Waltham, MA, USA) with 10% fetal bovine serum (FBS, Gibco), 1% HEPES (Sigma‐Aldrich, St. Louis, MO, USA), and 0.2% asparagine/arginine. Kuramochi cells were cultured in RPMI‐1640 (Gibco) with 10% FBS. Cells were maintained in T‐75 flasks (Corning, NY, USA) and passaged at 80% confluence using 0.1% EDTA in PBS (PAN Biotech, Aidenbach, Germany). Authentication of cell lines was performed via short tandem repeat (STR) profiling (Eurofins), and the absence of mycoplasma was verified regularly with Mycostrips^®^ (Invivogen).

### 2.3 Xenograft mouse experiment

Animal experiments were approved by the Government of Upper Bavaria (ROB) under licence Vet.: 2–22‐135 and conducted in accordance with animal protection guidelines (TUM University Hospital). For xenograft experiments, human OC cell lines (OV‐MZ‐6) transfected with either pRC‐RSV‐*KRT19* or empty vector were used. A total of 10^7^ tumour cells were intraperitoneally injected into eight‐week‐old female NU(NCr)‐*Foxn1* ^*nu*^ (athymic nude) mice, purchased from Charles River Laboratories. Mice were housed under pathogen‐free conditions in individually ventilated cages with no detectable specific pathogens. Animal housing and handling were performed in accordance with the German Animal Welfare Act (Tierschutzgesetz) and the Federation of European Laboratory Animal Science Associations (GV‐SOLAS) regulations. Mice were sacrificed upon signs of ascites and clinical symptoms. Euthanasia was performed using isoflurane anaesthesia followed by cervical dislocation. The peritoneal cavity was opened, and all visible tumour nodules were excised and weighed.

### 2.4 Patient samples

Tumour tissue specimens of 199 advanced‐stage HGSOC patients were collected between 1990 and 2012 from the biobank of the Department of Obstetrics and Gynaecology and the Institute of Pathology (part of the TUM University Hospital, Technical University of Munich, Germany). Ethics approval was granted by the local Ethics Committee (Faculty of Medicine, Technical University of Munich, Ismaninger Str. 22, 81 675 Munich, Germany; ethikkommission@mri.tum.de; project 491/17 S). Written informed consent was obtained from all patients. All patients underwent standard stage‐appropriate primary radical debulking surgery and platinum‐based adjuvant chemotherapy in combination with Taxol (Table S1). No patient received neoadjuvant treatment. Clinical factors recorded included age, ascites volume, residual tumour mass and FIGO staging (Table S1).

### 2.5 Stably transfected cell lines

Cells were seeded in 6‐well plates, grown to 70% confluence and transfected using Lipofectamine 3000 (Thermo Fisher Scientific, Waltham, MA, USA) with 2 or 4 μg of plasmid DNA. After six hours of incubation, the medium was replaced with FBS‐containing medium. Once confluent, cells were transferred to 10 cm dishes containing selection medium with 0.5 g·L^−1^ gentamicin (g418, Roth, Karlsruhe, Germany). Colonies were picked, and transfection was confirmed by reverse transcription‐quantitative PCR (RT‐qPCR) and western blot.

### 2.6 RNA extraction and RT‐qPCR

RNA was extracted using the NucleoSpin RNA Plus Kit (Macherey‐Nagel, Düren, Germany). Concentration and purity were measured with NanoPhotometer^®^ N60 (Implen, Munich, Germany). Reverse transcription was performed using RevertAid cDNA Kit (Thermo Fisher Scientific, Waltham, MA, USA). RT‐qPCR was conducted using TaqMan assays (Thermo Fisher Scientific, Waltham, MA, USA: *CDH1* (Hs01023895_m1), *CDH2* (Hs00983056_m1), *VIM* (Hs00958111_m1), *KRT19* (Hs00761767_m1)) and Blue Probe RT‐qPCR Kit (Biozym, Hessisch Oldendorf, Germany). Thermal conditions: 95 °C for 2 min; 40 cycles of 95 °C for 5 s, 60 °C for 25 s. Ct values were generally normalised to Hypoxanthine‐guanine phosphoribosyltransferase (HPRT) as an internal reference for differences in cDNA input and amplification efficiency.

### 2.7 Western blotting

Cells were lysed in radioimmunoprecipitation assay (RIPA) buffer supplemented with protease/phosphatase inhibitors (Roche, Mannheim, Germany). Protein was quantified via Pierce BCA assay (Thermo Fisher Scientific, Waltham, MA, USA). 40 μg protein was separated by 10% SDS/PAGE and transferred to a nitrocellulose membrane (Thermo Fisher Scientific, Waltham, MA, USA). Membranes were blocked in 5% milk in TBS‐T, incubated with primary antibodies overnight at 4 °C, and finally incubated with HRP‐conjugated secondary antibodies for 1 h. Detection of antibody‐stained proteins was performed using ECL (Millipore, Burlington, MA, USA), imaged on a ChemiDoc (Bio‐Rad, Hercules, CA, USA) and analysed with ImageJ. Antibodies used: anti‐KRT19 ab52625 (1 : 1000) (Abcam, Cambridge, UK), anti‐CDH1 sc‐59 905 (1 : 200), VIM sc‐6260 (1 : 500) (Santa Cruz, Biotechnology, Dallas, TX, USA), GAPDH MAB375 (1 : 10000); secondary antibodies: anti‐mouse G21040 (1 : 10000), anti‐rabbit G21234 (1 : 10000).

### 2.8 Chemoresistance and proliferation assay

Cell viability was assessed using the CellTiter‐Glo assay (Promega, Madison, WI, USA). Cells were treated with paclitaxel or carboplatin for 48 h (OV‐MZ‐6) or 72 h (Kuramochi). Luminescence was measured with a plate reader (BioTek, Winooski, VT, USA), normalised to untreated controls. Experiments were performed in triplicate.

### 2.9 Adhesion assay

96‐well plates were coated with fibronectin (2 μg·mL^−1^, Corning, NY, USA), vitronectin (2 μg·mL^−1^, Millipore, Burlington, MA, USA) or collagen IV (1 μg·mL^−1^, Sigma‐Aldrich, St. Louis, MO, USA) for one h and, thereafter, 2 × 10^4^ cells per well were seeded in adhesion buffer. After 90 min, wells were washed, and β‐hexosaminidase substrate (Sigma‐Aldrich, St. Louis, MO, USA) was added. Plates were incubated for 90 min, and the substrate reaction was stopped with EDTA/NaOH buffer. Absorbance was measured at 450 nm (Multiskan FC, Thermo Fisher Scientific). Data were normalised to KRT19‐low controls.

### 2.10 Wound scratch and motility assay

To assess cell migration, a wound scratch assay was performed using the CellWatcher system (Phio Scientific GmbH, Gilching, Germany). Briefly, cells were seeded in a 96‐well imaging plate at 90% confluence and cultured overnight in complete medium to allow attachment. After reaching the desired confluence of 100%, a uniform scratch was made across the monolayer. After scratching, the medium was replaced, and cells were imaged every 30 min for 63 h to quantify the gap closure and cell motility (in μm·h^−1^).

### 2.11 Trans‐well migration assay

Cell migration was assessed using a trans‐well migration assay with 8‐μm pore polycarbonate membrane inserts (Corning, NY, USA). Briefly, cells were serum‐starved for 12 h before the assay. A total of 5 × 10^4^ cells were resuspended in 500 μL of serum‐free medium and seeded into the upper chamber of the trans‐well insert. The lower chamber was filled with 1.5 mL of medium supplemented with either 20% FBS or 0.1% BSA in PBS.

After incubation at 37 °C with 5% CO_2_ for 4 h, non‐migrated cells were removed from the upper surface of the membrane. Migrated cells on the lower membrane surface were stained with one μg·mL^−1^ 4′,6‐diamidin‐2‐phenylindole (DAPI, Invitrogen, Carlsbad, CA, USA) for 15 min. Cell migration was quantified by imaging four fields per membrane under a light microscope using the QuPath software.

### 2.12 Spheroid formation

5 × 10^4^ cells per well were centrifuged to initiate spheroid formation and plated in 96‐well U‐bottom plates coated with poly‐HEMA (Sigma‐Aldrich, St. Louis, MO, USA). After 3–5 days, spheroids were transferred to poly‐HEMA‐coated 6‐well plates. Imaging was performed with the EVOS 2000 (Thermo Fisher Scientific, Waltham, MA, USA).

### 2.13 Isolation and cultivation of patient‐derived ascites cells

Ascites was processed immediately after surgery and cultured in selection medium as described before [19]. The cell suspension was incubated in the selection medium under standard culture conditions (37 °C, 5% CO_2_) for an initial adhesion period, allowing tumour cells to adhere while non‐adherent cells were washed away. After adhesion, tumour cells were maintained in complete medium with FBS and PenStrep (Gibco, Thermo Fisher Scientific, Waltham, MA, USA). Passaging was performed with 0.1% EDTA in PBS (PAN Biotech, Aidenbach, Germany).

### 2.14 Migration assay in 3D alginate matrix

A 3D migration assay was performed using alginate hydrogels to assess invasive capacity. Sterile 1.5% sodium alginate solution was prepared in PBS and supplemented with either 20% FBS (Gibco, Thermo Fisher Scientific, Waltham, MA, USA) to simulate directed migration, or no FBS to model random (undirected) migration. 500 μL of alginate solution was added per well of a 24‐well plate and allowed to solidify at room temperature.

Following gelation, 5 × 10^4^ cells in 500 μL of serum‐free medium were seeded onto each alginate gel. The cells were incubated for 24 h at 37 °C to allow for migration into the gel.

After incubation, the cell suspension above the gel was carefully removed, and the alginate surface was washed three times with PBS to eliminate non‐invaded cells. To recover invaded cells, 1 mL of 1% EDTA (PAN Biotech, Aidenbach, Germany) (w/v) in PBS was added to each well to dissolve the alginate matrix. Plates were incubated at 37 °C until the gel was completely liquefied. The resulting cell suspension was collected, centrifuged, and the pellet was resuspended in a defined volume of 200 μL PBS. For quantification, an aliquot of the cell suspension was mixed 1 : 1 with 0.1% Trypan Blue solution (Sigma Aldrich, Merck KGaA, Darmstadt, Deutschland), and viable (unstained) cells were counted manually using a Neubauer haemocytometer.

### 2.15 Next‐generation sequencing (NGS) analysis of cell line DNA

Genomic DNA was isolated from OV‐MZ‐6 and Kuramochi cells. NGS and data analysis were performed on an Illumina sequencing platform using the TruRisk^®^ Sequencing Panel (Illumina, San Diego, CA, USA), a targeted NGS assay designed to detect clinically relevant genomic alterations in breast and ovarian cancer patients. Target enrichment was performed using the TruSight Rapid Capture System (Illumina, San Diego, CA, USA). The data were analysed for exonic copy number variations (CNVs) and single‐ and multi‐nucleotide variants in coding exons and adjacent intronic sequences (± 20 bp).

### 2.16 Proteomic gene set enrichment analysis (GSEA)

Proteomic profiling was performed on OV‐MZ‐6 cell lines either overexpressing KRT19 or transduced with an empty vector control. Briefly, protein lysates were digested using the S‐Trap spin column. Afterwards, samples were 16plex TMT‐labelled and pre‐fractionated on an Agilent HPLC system. MS samples were measured on a Q Exactive Plus mass spectrometer. MS files were analysed using MaxQuant and further processed for statistical analysis using Limma. The resulting protein expression data were normalised and analysed using a gene set enrichment analysis framework to identify significantly altered signalling pathways and biological processes.

GSEA was conducted using predefined hallmark gene sets and a sign(log₂ fold change) × −log_10_(p)‐ based ranking metric. The analysis was performed with Python. The analysis focused on migration‐associated signalling pathways, specifically Notch, Wnt/β‐catenin, RAC1, and general EMT markers. Protein data were mapped to predefined protein groups (gene sets) obtained from publicly available databases, such as the MSigDB Hallmark gene sets.

### 2.17 Bioinformatics

RNA expression data for KRT19 in normal versus tumour ovarian tissue were obtained from The Cancer Genome Atlas (TCGA) database via the online bioinformatic tool UCSC Xena [20] (accessed in November 2024). The dataset included RNA sequencing data from 88 normal ovarian tissues and 426 high‐grade serous ovarian carcinoma (HGSOC) samples. This was consistently observed across multiple normalisation methods, including STAR counts and TPM. *In silico* analysis of overall survival in ovarian cancer patients, stratified by *KRT19* expression levels and treatment status, was conducted using the Kaplan–Meier Plotter database [21], including data from Gene Expression Omnibus (GEO), Genotype‐Tissue Expression (GTEX), TCGA, and Therapeutically Applicable Research to Generate Effective Treatments (TARGET) databases (accessed in January 2025). This analysis utilised data from 1100 serous ovarian cancer cases, which were further filtered based on tumour grade and therapeutic interventions, and subsequently divided into appropriate cohorts for comparative survival analysis.

### 2.18 Software and statistics

All experiments were conducted in at least three biological replicates. Data were analysed and visualised using the GraphPad Prism software (version 8.2.1) and are presented as mean ± standard error of the mean (SEM). Semi‐quantitative analysis of western blot signals was performed using the imagej software (ImageJ.exe), with subsequent data visualisation in graphpad prism. Quantification of cell migration in transwell assays was performed using QuPath (version 0.5.1), followed by statistical analysis in GraphPad Prism. Kaplan–Meier estimates were performed using the log‐rank test, and univariate and multivariable analyses were performed using Cox proportional hazards regression. An unpaired *t*‐test was applied to compare the two groups for parametric data. When data were nonparametric, the Mann–Whitney *U*‐test was used. Two‐way ANOVA was used to analyse wound healing (gap closure) in scratch assays. Statistical significance was defined as *P* < 0.05.

### 2.19 Cut‐off determination

Cut‐off values for dichotomising KRT19 expression were determined using the X‐tile algorithm [22], identifying the optimal threshold based on survival outcomes. To ensure robustness, alternative cut‐offs (median, tertiles, and continuous modelling) were also evaluated, yielding comparable prognostic results. For *in silico* analyses based on online databases (e.g. Kaplan–Meier Plotter), cut‐off determination followed the internal calculation procedures implemented by the respective platforms.

## 3 Results

### 3.1 KRT19 is overexpressed in HGSOC and correlates with decreased overall survival

**Fig. 1:**
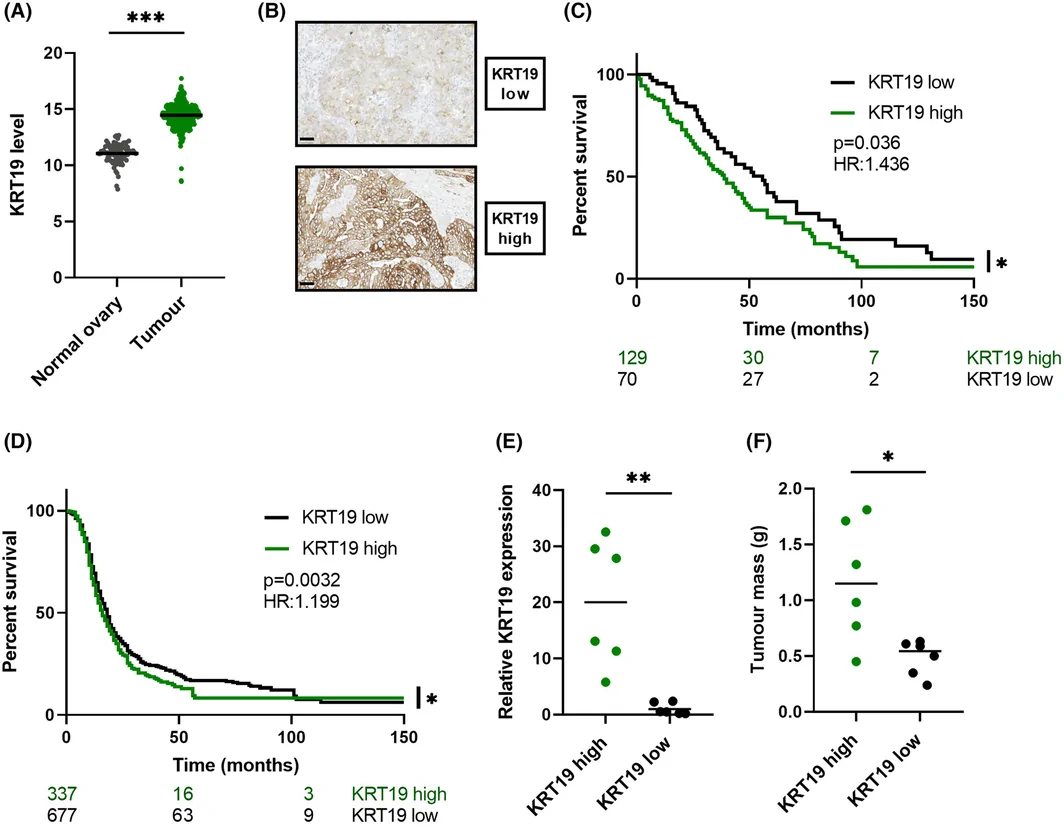
KRT19 is overexpressed in high‐grade serous ovarian cancer (HGSOC) and is associated with poor overall survival and increased tumour burden. (A) Comparison of KRT19 expression in healthy ovary (*n* = 88) vs. primary tumour from HGSOC patients (*n* = 426; data derived from TCGA database). (B) Representative immunohistochemistry images depicting high or low Keratin 19 (KRT19) levels in primary HGSOC tumour tissues (cohort see Table 1); scale bar 50 μm. (C, D) Overall survival of HGSOC patients stratified by KRT19 expression. (C) Patients treated at the MRI TUM University Hospital between 1990 and 2014 (low *n* = 70, high *n* = 129; cohort see Table 1). High‐ and low‐KRT19 expression groups were defined using an optimised cut‐off at the 33rd percentile of expression values, as determined by the X‐tile algorithm. (D) Data sourced from the Kaplan–Meier Plotter program using GEO, GTEX, TCGA and TARGET databases as of November 2024 (low *n* = 677, high *n* = 337). For analyses based on Kaplan–Meier Plotter datasets, high‐ and low‐expression groups were defined according to the cut‐off values provided in the analysis platform. Statistical significance for Kaplan–Meier plots was calculated with the log‐rank test. The (E, F) Xenograft mouse model was established by *intraperitoneal* injection of OV‐MZ‐6 KRT19+ (KRT19 high) or empty vector (KRT19 low) cells into nude mice (KRT19 high, *n* = 6; KRT19 low, *n* = 6). (E) Validation of stable KRT19 overexpression in primary tumour tissue via RT‐qPCR. Ct values were normalised to the mean KRT19 mRNA expression of the empty vector cohort. (F) Tumour mass in KRT19 high‐ vs low‐expressing mice; *P < 0.05, **P < 0.01, ***P < 0.001; (A) median, (C–F) error bars ± SEM. Statistical significance for A, E and F was determined with an unpaired *t*‐test, and for C and D with the log‐rank test.

Analysis of publicly available RNA data [20] revealed significant *KRT19* mRNA overexpression in primary HGSOC tumours (*n* = 426) versus normal ovarian tissue (*n* = 88) (Fig. 1A). Immunohistochemistry in a well‐characterised, homogenous patient cohort (Table S1) confirmed tumour‐specific, cytoplasmic KRT19 expression (Fig. 1B).

Kaplan–Meier analysis linked high KRT19 levels to reduced overall survival (Fig. 1C; hazard ratio: 1.436, *P* = 0.036), and multivariable Cox regression accounting for age, ascites volume, residual tumour burden, and FIGO staging identified KRT19 as an independent prognostic factor (Table 1; *P* = 0.002; hazard ratio: 2.41). However, no significant effect of KRT19 on progression‐free survival was observed.

**Table 1:** Multivariable Cox regression analysis.

Clinicopathological parameters	PFS			OS		
	No^a^	HR (95% CI)^b^	*P*	No^a^	HR (95% CI)^b^	*P*
Age			0.644			0.356
≤ 60 years	40	1		41	1	
> 60 years	42	1.13 (0.67–1.93)		50	1.24 (0.79–1.95)	
Ascitic volume			0.076			0.408
≤ 500 mL	19	1		22	1	
> 500 ml	63	1.67 (0.95–2.96)		69	1.14 (0.75–2.04)	
Residual tumour			**0.013**			**<0.001**
≤ 10 mm	50	1		56	1	
> 10 mm	32	2.04 (1.16–3.59)		35	2.41 (1.42–4.09)	
FIGO stage			0.861			0.118
III	55	1		60	1	
IV	26	1.055 (0.58–1.93)		30	1.48 (0.91–2.43)	
KRT19 protein^c^			0.060			**0.002**
Low	25	1		26	1	
High	57	1.81 (0.96–3.39)		65	2.41 (1.40–4.15)	

Chi‐square test, significant *P*‐values (*P* < 0.05) are indicated in bold.

a Number of patients.

b HR: hazard ratio (CI: confidence interval) of univariate Cox regression analysis.

c Dichotomised into low and high levels by the 33rd percentile.

External datasets (GEO, GTEX, TCGA, TARGET) supported the association between high *KRT19* expression and poor prognosis (Fig. 1D) [21]. Further, *in vivo* xenografts using OV‐MZ‐6 cells overexpressing KRT19 (Fig. 1E) developed significantly greater tumour burden than controls (*P* = 0.03, Fig. 1F), though the peritoneal spread pattern remained similar.

These findings suggest that KRT19 is not only a negative prognostic marker in HGSOC but also promotes tumour progression.

### 3.2 KRT19 overexpression in HGSOC cell lines

**Fig. 2:**
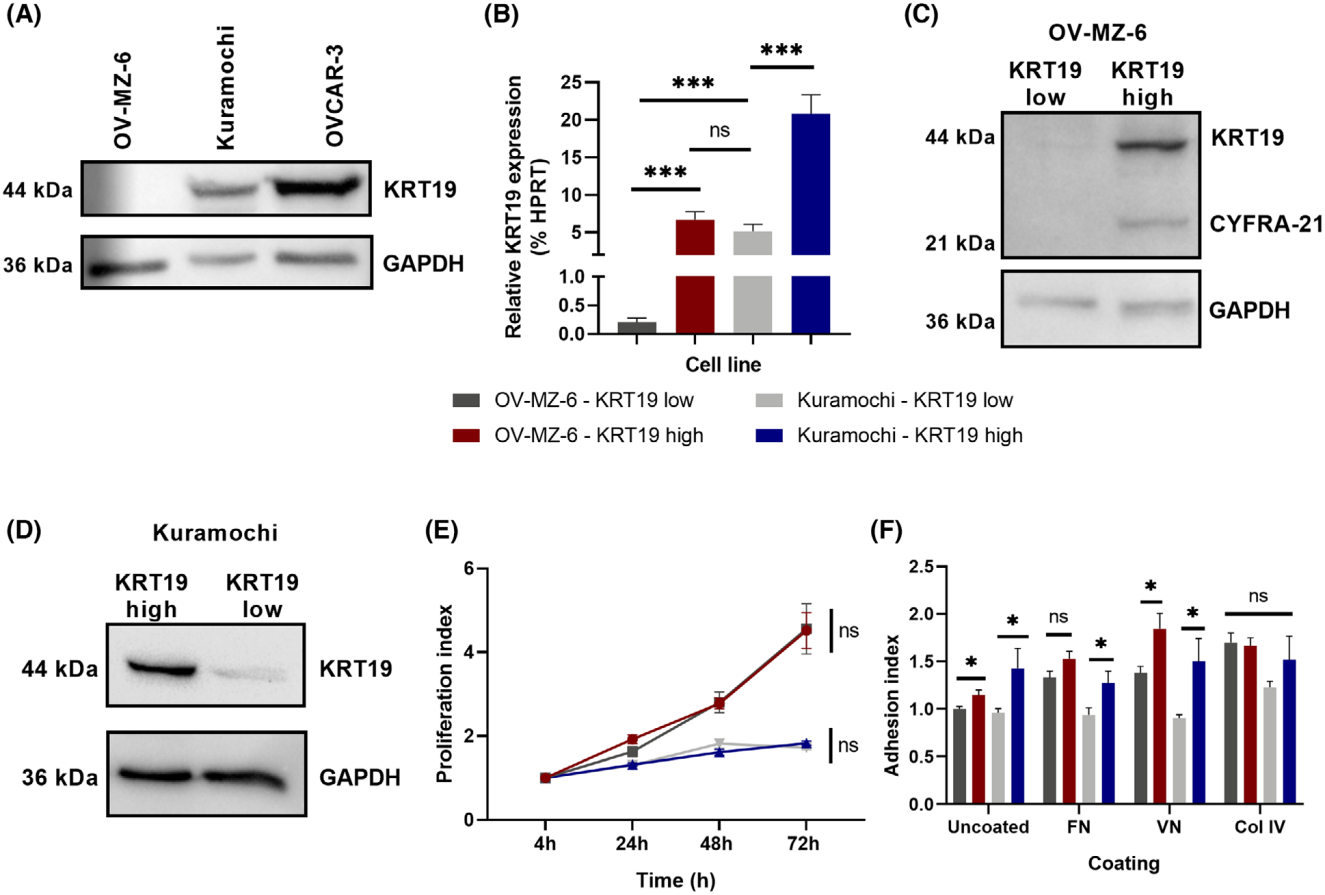
KRT19 enhances adhesion without affecting proliferation in high‐grade serous ovarian cancer (HGSOC) OV‐MZ‐6 and Kuramochi cell lines. (A) Comparison of Keratin 19 (KRT19) protein expression in wild‐type OV‐MZ‐6, Kuramochi and OVCAR‐3 cell lines via western blot analysis (*n* = 2) (also see Fig. S2A). (B) Cell lines were transfected with either the empty vector pRC‐RSV (RSV) or the vector harbouring the KRT19 coding region. Elevated KRT19 expression was confirmed by RT‐qPCR (both at least *n* = 3). (C, D) KRT19 overexpression on the protein level was validated by western blot analysis (OV‐MZ‐6 *n* = 3, Kuramochi *n* = 4) (also see Fig. S2B,C). (E) Proliferative activity of KRT19 high vs. KRT19 low cells was assessed using the Cell Titer Glo assay (both *n* = 4). (F) Cell adhesive capacity was evaluated by a modified Landegren assay. Cells were seeded in 96‐well plates and allowed to adhere for 90 min in fibronectin (FN)‐, vitronectin (VN)‐ or collagen IV (Col IV)‐coated or uncoated wells. Fold change was given relative to uncoated wells bearing KRT19 low‐expressing cells (*n* = 3); ns, not significant; *P* > 0.05; *P < 0.05; ***P < 0.001; error bars ± SEM. Statistical significance for B, E, and F was determined with an unpaired *t*‐test.

To investigate the cellular mechanisms underlying KRT19‐driven aggressiveness in HGSOC, we overexpressed KRT19 in two cell lines. Despite its structural role, KRT19 overexpression did not induce any apparent changes in cell morphology (Fig. S1A,B). Cell lines with low to moderate KRT19 expression were chosen (OV‐MZ‐6 and Kuramochi) compared to the KRT19 high‐expressing OVCAR‐3 cell line (Fig. 2A, Fig. S2A). Stable overexpression was confirmed by RT‐qPCR (Fig. 2B) and validated at the protein level by western blot, including detection of the 21 kDa KRT19‐derived fragment CYFRA‐21 (Fig. 2C,D, Fig. S2B,C).

### 3.3 KRT19 increases cell adhesion and directional migration *in vitro*

To investigate whether KRT19 directly affects tumour growth, proliferation was assessed in Kuramochi and OV‐MZ‐6 cells, which differ 3‐fold in baseline growth rates. KRT19 overexpression did not impact proliferation within 72 h in either line (Fig. 2E).

To determine whether KRT19 may influence metastatic processes, effects on cell adhesion and migration were examined. Adhesion assays showed enhanced binding of KRT19‐overexpressing OV‐MZ‐6 cells to uncoated surfaces and extracellular matrix (ECM) proteins, with up to a 50% increase on vitronectin and a similar trend on fibronectin, but no effect on collagen IV (Fig. 2F).

**Fig. 3:**
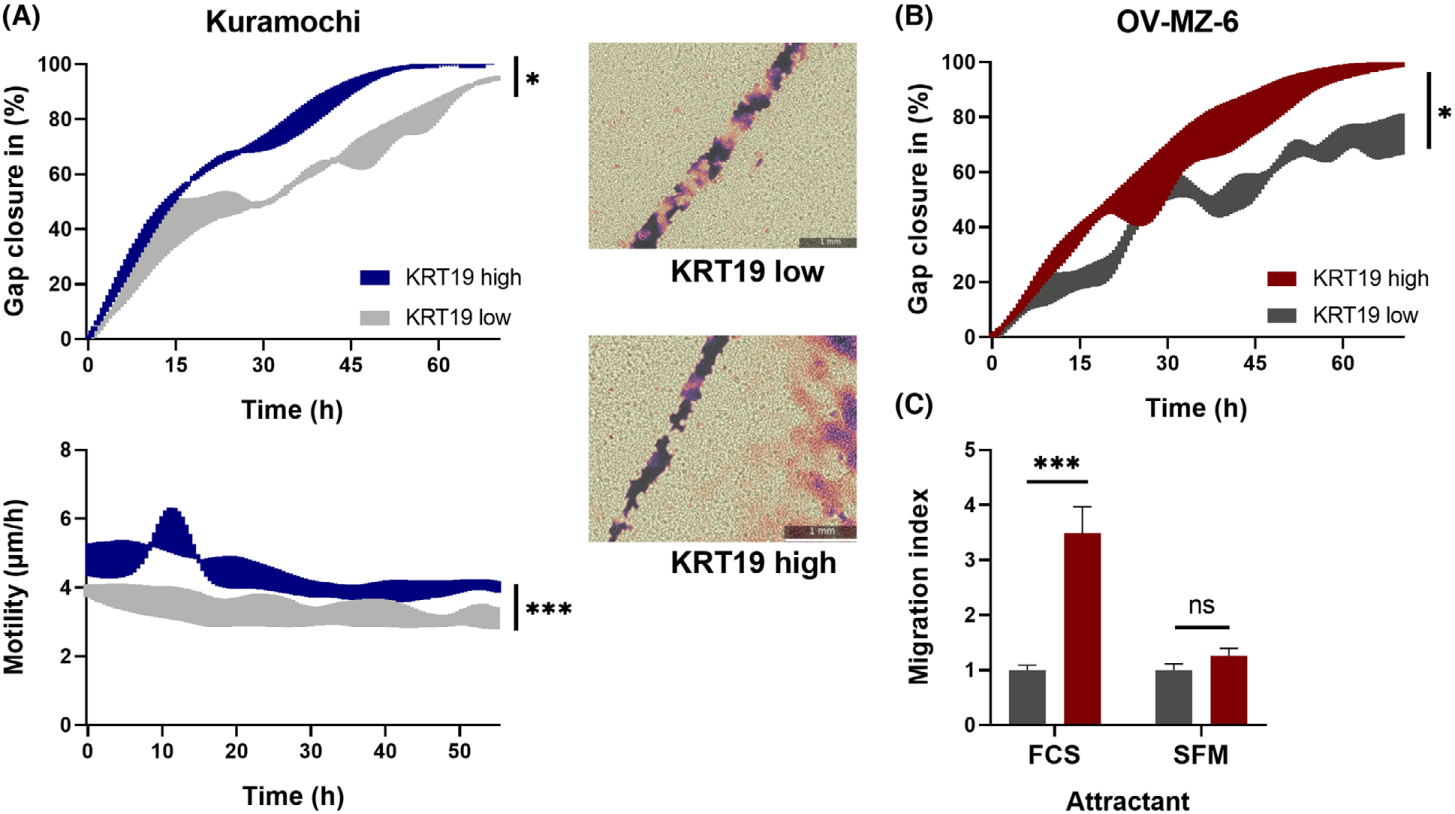
Keratin 19 (KRT19) leads to increased migration and motility capabilities *in vitro*. (A and B) Wound scratch assays were performed with Kuramochi (A) and OV‐MZ‐6 (B) KRT19^+^ vs control cell lines and analysed with the Cell Watcher System (PHIO Scientific GmbH) (both *n* = 3). Representative pictures of Kuramochi KRT19^+^ vs. control cells show differences in the gap closure efficiency after 25.5 h. Yellow dots represent cells, magenta‐stained dots represent moving cells. Motility differences in KRT19 high‐ vs. low‐expressing cells were evaluated by the velocity of cells migrating into the gap of the wound scratch, analysed with the Cell Watcher; scale bar 1 mm (*n* = 3). (C) Additionally, directional migration activity of OV‐MZ‐6 KRT19+ vs. control was evaluated via a trans‐well assay, with cells migrating towards either FBS or serum‐free medium (SFM). The nuclei of migrated cells were stained with DAPI and counted using QuPath (*n* = 4). Representative staining pictures see Fig. S5B; ns, not significant; *P > 0.05; ***P < 0.001; error bars ± SEM. Statistical significance for A, B, and C was determined with a one‐way ANOVA test.

Beyond adhesion, cell migration plays a crucial role in metastasis by facilitating the dissemination of tumour cells from their site of origin into the bloodstream and distant tissues. Wound‐healing assays revealed a 30% reduction in gap closure upon KRT19 overexpression (Fig. 3A,B), while motility remained significantly higher (Fig. 3A), supporting a promigratory role of KRT19. Directional migration assays with the OV‐MZ‐6 cell line further confirmed a 3.5‐fold increase in chemotactic response towards 20% in KRT19^+^ cells (Fig. 3C).

Although proliferation was unaffected, these results indicate that KRT19 promotes key metastatic behaviours, including enhanced, directed migration and altered adhesion, which may facilitate their dissemination and contribute to metastasis.

### 3.4 KRT19 expression correlates with a more epithelial cell phenotype

The ability of epithelial cells to transition into a motile mesenchymal phenotype, and vice versa, is central to cancer progression and metastasis. The impact of KRT19 on EMT marker expression was analysed both at the RNA and protein levels.

**Fig. 4:**
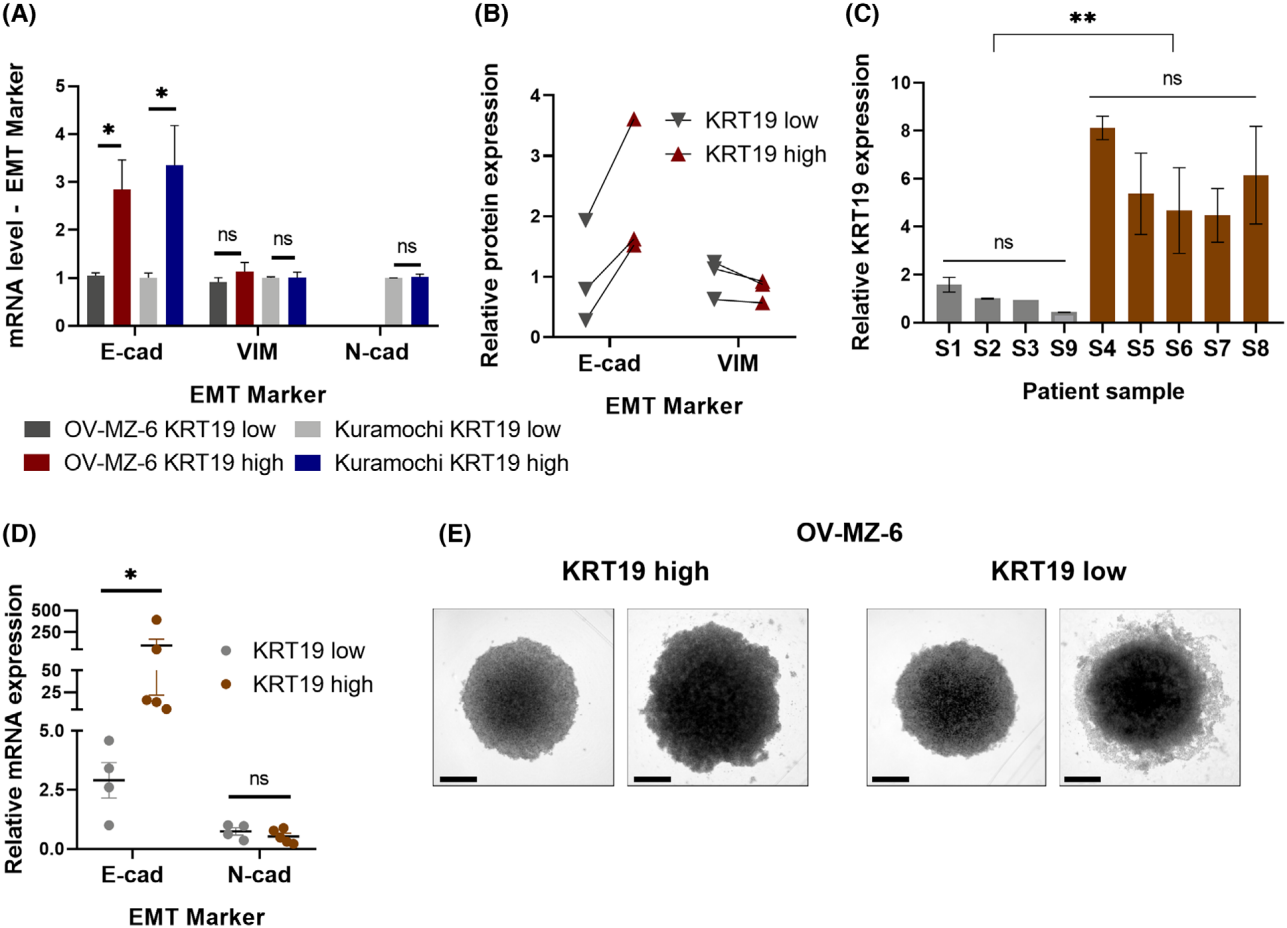
Keratin 19 (KRT19) leads to a shift towards an epithelial phenotype. (A–D) Alterations in epithelial‐mesenchymal transition (EMT) marker expression were analysed as a function of KRT19 expression in OV‐MZ‐6 and Kuramochi cell lines (epithelial marker: E‐cadherin (E‐cad); mesenchymal marker: vimentin (VIM) and N‐cadherin (N‐cad)). (A) *In vitro* evaluation on the transcriptional level (RT‐qPCR) for both cell lines (all *n* = 4). N‐cadherin was not detectable in the OV‐MZ‐6 cell line. (B) *In vitro* evaluation on the protein level (western blot assay) for OV‐MZ‐6 cell line (each marker *n* = 3). Due to low‐expression levels, visible bands were analysed via ImageJ. N‐cadherin was undetectable due to its low‐expression levels (see A). For western blots, also see Fig. S3A,B. (C) Tumour cells were isolated from the ascites of nine patients, and KRT19 mRNA expression was measured *ex vivo*. (D) In KRT‐high vs. KRT‐low cells, isolated from different patient samples (see C), E‐ and N‐cadherin mRNA expression was measured via RT‐qPCR. E Representative pictures of spheroids formed by OV‐MZ‐6 KRT19^+^ cells vs. empty vector cells after 10 days; scale bar 50 μm (*n* = 8). ns, not significant; *P* > 0.05; *P < 0.05; **P < 0.01; error bars ± SEM. Statistical significance for A, C, and D was determined with an unpaired *t*‐test.

*In vitro*, KRT19 overexpression led to a 3 to 4‐fold increase in E‐cadherin (*CDH1*) mRNA, while neural cadherin (N‐cadherin, *CDH2*) and vimentin (*VIM*) levels remained unchanged (Fig. 4A). Western blot analysis confirmed a ~ 2‐fold increase in E‐cadherin protein levels in KRT19^+^ cells, with no change in vimentin (Fig. 4B, also see Fig. S3A,B).

Next, we determined *KRT19*, *CDH1* and *CDH2* mRNA in ascites tumour cells (ATCs) from ascites of nine HGSOC patients, stratified into KRT19‐low (*n* = 4) and KRT19‐high (*n* = 5) groups (Fig. 4C). Notably, ATCs from the KRT19‐high group showed nearly a 25‐fold increase in *CDH1*, while mesenchymal markers remained unchanged (Fig. 4D), mirroring the *in vitro* findings.

These results consistently associate high KRT19 expression with an epithelial phenotype in both cell lines and *ex vivo* models.

To explore whether this phenotype confers a functional advantage to ATCs, we analysed spheroid growth of KRT19^+^ OV‐MZ‐6 cells. After ten days, KRT19^+^ spheroids exhibited a compact structure with well‐preserved integrity, unlike KRT19‐low spheroids, which showed disrupted cell–cell junctions and disorganised outer cell layers (Fig. 4E). These findings suggest that elevated KRT19 expression enhances spheroid cohesion by reinforcing epithelial traits of mesenchymal cells, potentially promoting the survival of ATCs in ascitic fluid.

**Fig. 5:**
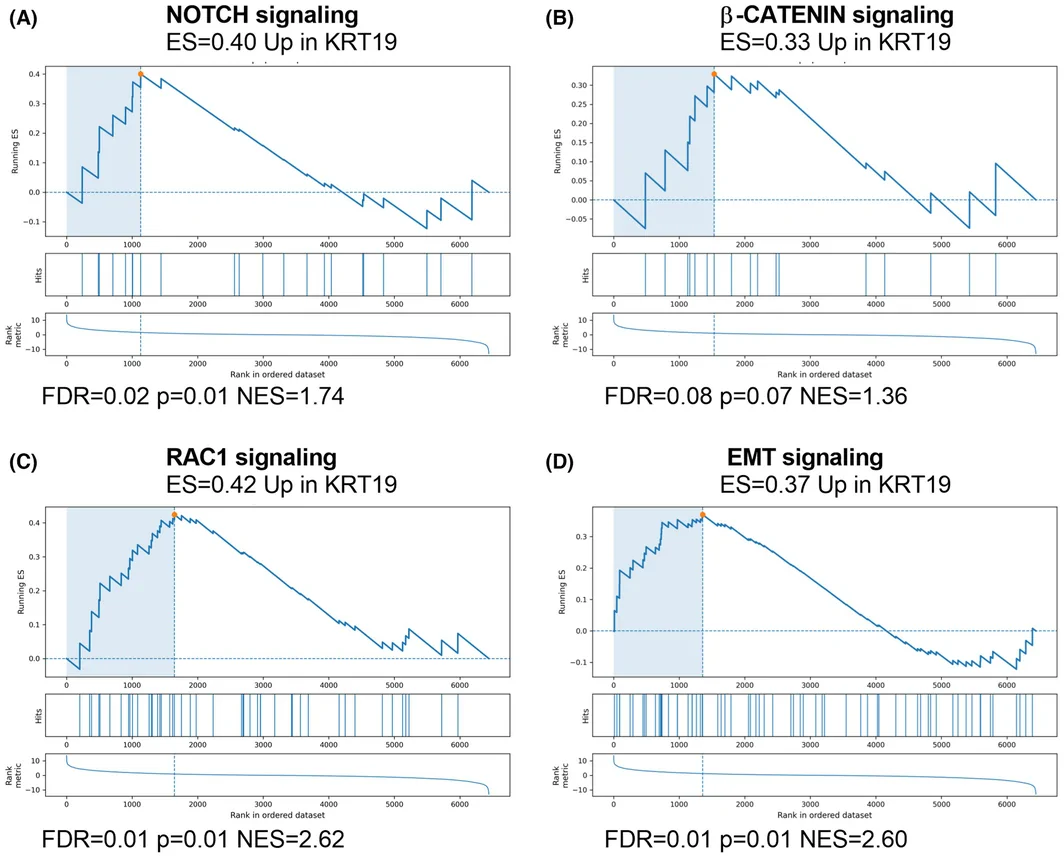
Gene Set Enrichment Analysis (GSEA) of migration‐ and EMT‐related pathways in OV‐MZ‐6 cells overexpressing KRT19 compared to empty vector controls. (A) GSEA for upregulated genes associated with the NOTCH pathway. (B) GSEA for upregulated genes related to the WNT/Catenin pathway. (C) GSEA for upregulated genes associated with the RAC1 pathway. (D) GSEA for upregulated genes linked to EMT‐associated pathways. Enrichment Score (ES) indicates the degree to which a gene set is overrepresented at the top or bottom of the ranked list of genes. The Normalised Enrichment Score (NES) accounts for differences in gene set size and variation across datasets, allowing for comparisons between gene sets. The *P*‐value reflects the statistical significance of the enrichment, calculated based on permutation testing.

To explore potential mechanisms underlying the altered EMP and the observed increase in migration and motility induced by KRT19, we performed a proteomic analysis followed by GSEA. Specifically, we focused on key signalling pathways implicated in EMT and cell migration: Notch, β‐catenin and RAC1 (Fig. 5A–D). Our analysis revealed a consistent upregulation of these pathways in KRT19‐overexpressing cells compared to controls. Enrichment scores ranged between 0.33 and 0.42, indicating moderate but significant pathway activation associated with KRT19 expression.

### 3.5 KRT19 increases the chemoresistance towards first‐line pharmacotherapy

Chemoresistance remains a major clinical challenge in OC and has been linked to epithelial–mesenchymal characteristics of tumour cells [23]. To investigate the role of KRT19 in this context, *in vitro* cytotoxicity assays were performed using OV‐MZ‐6 and Kuramochi cell lines treated with the first‐line chemotherapeutic agents paclitaxel (Taxol) and carboplatin. Dose–response experiments were carried out to define appropriate concentrations (see Fig. S4A,B).

**Fig. 6:**
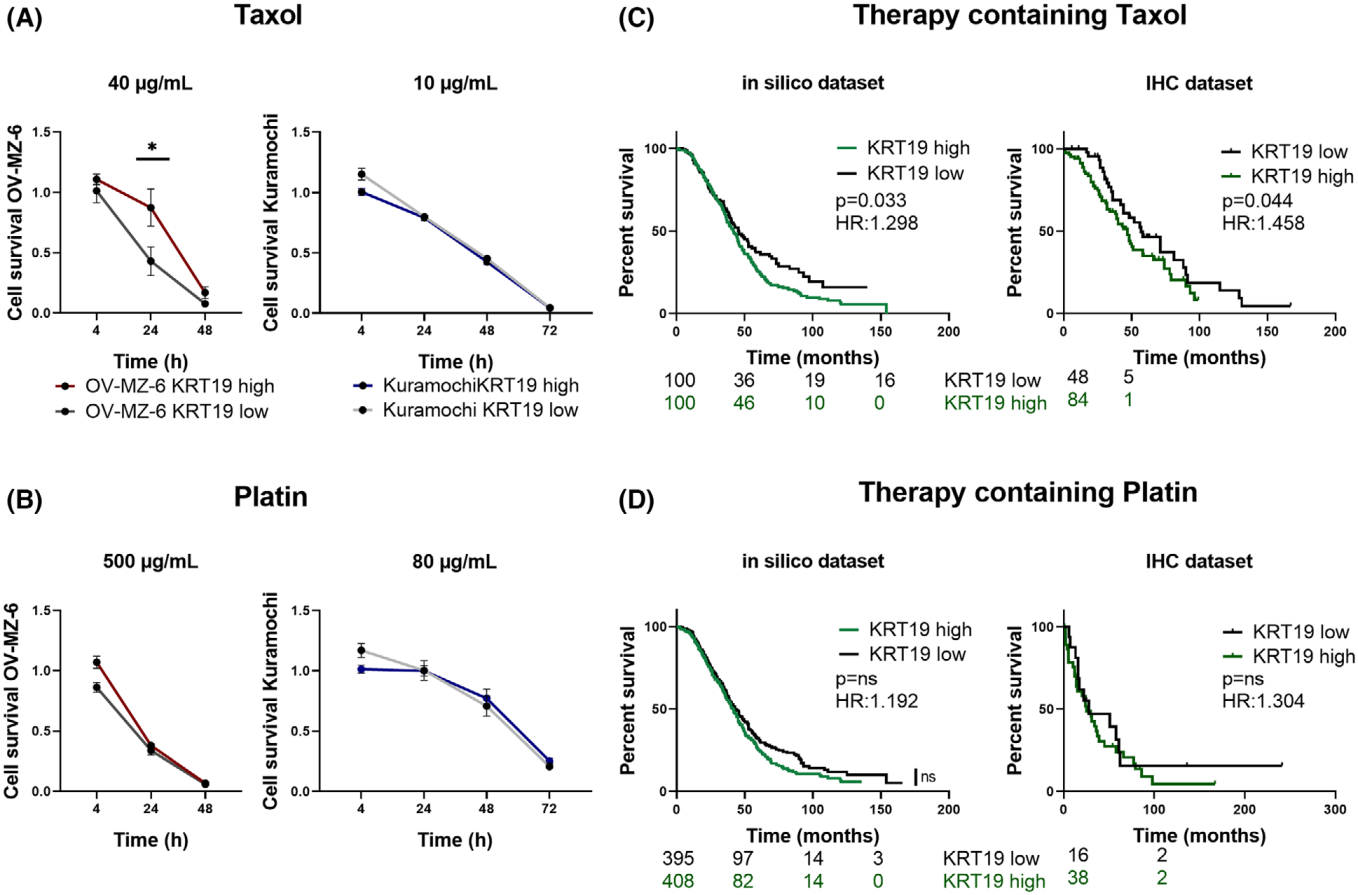
Keratin 19 (KRT19) may lead to increased chemoresistance in first‐line pharmacotherapy. (A and B) OV‐MZ‐6, and Kuramochi cell lines were treated with (A) Taxol or (B) platinum at a suitable concentration. Depending on the growth rate, the experiment lasted 48–72 h (at least *n* = 3 for all cell lines). Cell survival at each time point was determined via the CellTiter Glo assay. (C, D) Overall survival of HGSOC patients receiving (C) Taxol + carboplatin (*in silico*: KRT19 low *n* = 100, high *n* = 100; IHC: KRT19 low *n* = 48, high *n* = 84) or (D) only carboplatin (*in silico*: KRT19 low *n* = 395, high *n* = 408; IHC: KRT19 low *n* = 16, high *n* = 38) containing pharmacotherapy stratified by KRT19 expression. Data are from the Kaplan–Meier Plotter program using GEO, GTEX, TCGA, and TARGET databases, as of February 2025 (low *n* = 697, high *n* = 354) and subgroup analysis of the ovarian cancer cohort of this study (IHC cohort); ns, not significant; *P* > 0.05; *P < 0.05; ***P < 0.001; error bars ± SEM. Statistical significance was determined with an unpaired *t*‐test.

KRT19 overexpression significantly enhanced resistance towards Taxol in OV‐MZ‐6 cells, doubling survival rates within 24 h compared to controls (Fig. 6A). However, KRT19 did not affect OV‐MZ‐6 cells treated with carboplatin (Fig. 6B). In Kuramochi cells, KRT19 had no impact on response to either agent (Fig. 6A,B). To explore the clinical relevance of these findings, overall survival was analysed in HGSOC patients (FIGO III‐IV) receiving platinum‐based compounds with (Fig. 6C) or without (Fig. 6D) additional Taxol treatment via Kaplan–Meier estimation [21]. Patients with high KRT19 expression exhibited significantly reduced overall survival in the paclitaxel‐treated group (*P* < 0.05), while this effect was diminished in the platinum‐only group (Fig. 6C,D). In line with the *in silico* analysis, a subgroup analysis of our immunohistochemistry data stratified by treatment regime (platinum monotherapy versus platinum + Taxol combination therapy) was performed. In patients treated with carboplatin alone, KRT19 lost its prognostic significance with respect to worse outcome. In contrast, in patients treated with carboplatin in combination with Taxol, high KRT19 expression remained significantly associated with reduced overall survival. These findings suggest that KRT19 may specifically contribute to paclitaxel resistance in HGSOC, as observed *in vitro* in OV‐MZ‐6 cells.

## 4 Discussion

### 4.1 KRT19 correlates with decreased overall survival and increased tumour progression

The role of KRT19 as a prognostic factor varies across tumour types, acting as either a tumour suppressor or a promoter depending on the tumour type [13, 24]. As HGSOC is the most common OC subtype and accounts for up to 80% of OC‐related deaths, we focused on this subtype. KRT19 mRNA is upregulated in ovarian cancer tissue compared to healthy ovarian tissue. However, this comparison does not fully reflect the biology of ovarian cancer initiation, as healthy ovarian tissue comprises multiple cell types and compartments that are not directly involved in carcinogenesis. To address this limitation, an *in silico* analysis of benign ovarian lesions and healthy ovaries was performed, revealing no changes in KRT19 expression in non‐malignant tissue (see Fig. S6). These findings further support a carcinogenesis‐associated upregulation of KRT19.

We found that high KRT19 expression correlated with poor survival in a homogeneous HGSOC cohort, consistent with other studies in HGSOC and lung squamous cell carcinoma [16, 25, 26]. We confirmed this association both at the protein level in 199 HGSOC patients and by *in silico* mRNA analysis. Moreover, KRT19‐overexpressing xenograft tumours exhibited increased tumour burden compared to controls.

It has been suggested that the contrasting effects of KRT19 on tumour progression may be mediated by differential regulation of pathways, such as ß‐catenin, which impacts EMP [24, 27, 28]. For example, Saha et al. [24] showed that KRT19 knockout reduced proliferation, migration, and spheroid formation in colon carcinoma cells but increased these traits in breast cancer lines. These discrepancies suggest subtle, context‐dependent regulatory roles of KRT19. Given this, we aimed to clarify KRT19's specific effects on key processes, including proliferation, migration and EMT in HGSOC.

### 4.2 KRT19 supports metastatic processes

Metastasis remains the leading cause of cancer‐related death and a major therapeutic challenge [29]. In HGSOC, the absence of robust mechanical barriers facilitates direct shedding of tumour cells into the peritoneal cavity, contributing to its particularly high metastatic potential and rapid tumour dissemination [2, 26, 30]. Understanding these mechanisms is critical for identifying effective biomarkers or therapeutic strategies. Therefore, we investigated the effects of KRT19 expression in the HGSOC cell lines Kuramochi and OV‐MZ‐6 on proliferation and metastasis‐relevant processes. These cell models were selected based on their differing wild‐type KRT19 expression profiles, allowing us to minimise the likelihood of off‐target or random transfection‐induced effects. The OV‐MZ‐6 cell line exhibits negligible endogenous KRT19 levels, resulting in a substantial relative increase in expression upon KRT19 overexpression. To provide a more physiologically comparable model, the Kuramochi cell line was also included. A notable limitation, however, is the absence of a loss‐of‐function model in our experimental design, which prevents a direct comparison with a scenario lacking functional KRT19. To assess the credibility of the observed effects despite the missing loss‐of‐function model, we additionally employed complementary *in silico, in vivo, and ex vivo* models. Nevertheless, this limitation should be taken into account when interpreting the *in vitro* findings. We found that KRT19 did not affect proliferation in either cell line. This contrasts with previous reports for other cancer cell lines, such as the breast cancer cell line MCF7 [24, 27]. This discrepancy supports the hypothesis that tumour cells undergo a ‘grow or go decision’ based on their invasive characteristics, as sustaining both proliferation and migration simultaneously would impose excessive metabolic demands. Cell migration requires substantial cellular energy to drive actomyosin‐based contractility and actin polymerisation. Recent studies have highlighted that altered cancer mechano‐signalling enables tumour cells to maintain high metabolic activity during metastasis, particularly when navigating microenvironments with varying stiffness [12]. The metastasis cascade comprises several critical steps: (1) local migration through enhanced motility and migration capabilities, (2) survival in circulation, (3) arrest at the target organ and (4) successful colonisation at the metastatic site [12, 31].

Focusing on the first step—local migration—we observed that KRT19 enhances migratory capacity in both OV‐MZ‐6 and Kuramochi cell lines. In a wound‐healing assay, KRT19+ cells exhibited significantly faster gap closure and increased motility compared to controls. Furthermore, OV‐MZ‐6 KRT19^+^ cells displayed a markedly enhanced ability for directional migration. The role of KRT19 as an intermediate filament in migration and potentially invasion may be linked to its known function in supporting mechanical plasticity and facilitating cytoskeletal remodelling—both essential processes for cell migration and motility. This implies that KRT19 plays a pivotal role in promoting the invasive behaviour of tumour cells by enhancing the structural adaptability required for metastatic progression [12, 32]. To migrate, cells must extend invadopodia, which form a network of intermediate filaments and microtubules, to perform translocation by combining protrusion and adhesion at the front of the cell with contraction and de‐adhesion at the back of the cell [12, 33, 34]. The observed increase in motility, migration and adhesion provides strong evidence for a tumour microenvironment more permissive to metastasis under KRT19 expression. However, it does not constitute definitive proof, as invasive behaviour was not directly assessed. To address this limitation, we performed initial 3D migration assays using an alginate‐based model, which provided strong preliminary evidence that KRT19 expression promotes enhanced directional invasive behaviour (Fig. S5A).

Adhesion is similarly vital not only for migration but also for the second step in ATC sphere formation. These cell aggregates must prevent anoikis and mediate reattachment to the metastatic site and colonisation [24, 29]. Intercellular adhesion, on the one hand, and adhesion to the ECM, on the other, are relevant for this purpose.

It is already known that KRT19 knockout leads to weakened intercellular adhesion in 2D cell culture of breast cancer. Alsharif et al. [27] also showed that KRT19 knockout in the MCF‐7 cell line reduced 3D spheroid formation. Our findings further support the role of KRT19 in intercellular adhesion, as we observed that sphere formation, density, and structural integrity were maintained in KRT19^+^ OV‐MZ‐6 cells after 10 days of growth. In contrast, in the control cell line, the outer cell layer was loose and dispersed into the medium. Given that detached cells typically undergo anoikis [29], it is plausible that increased KRT19 expression enhances intercellular adhesion, thereby improving ATC survival during metastasis [35]. Research further hypothesises that the ability to form spheroids could even lead to increased integrin‐mediated invasiveness on human mesothelial monolayers and chemoresistance [35, 36].

Furthermore, our findings indicate that KRT19 significantly influences adhesion to various ECM proteins and to uncoated polystyrene surfaces. Notably, we observed an increased adhesion towards the ECM proteins vitronectin and fibronectin in both Kuramochi KRT19^+^ and OV‐MZ‐6 KRT19^+^ cell lines compared to the control. This suggests that KRT19 enhances cell reattachment to the extracellular matrix, potentially accelerating the formation of secondary metastases.

In summary, our *in vitro* data demonstrate that KRT19 promotes cell migration, increases intercellular adhesion—thereby supporting the survival of unattached cells—and enhances adhesion to ECM surfaces, facilitating reattachment to secondary tissues. Since these are all critical factors in the metastatic cascade, our findings strongly suggest that KRT19 not only contributes to overall tumour burden but may also actively promote dissemination and metastasis. Linking these findings to the clinical endpoints, KRT19 appears to have little influence on early tumour dynamics, which are largely driven by proliferative processes, and therefore only marginally affects progression‐free survival. In contrast, its pronounced impact on later stages of tumour evolution—characterised by enhanced metastatic behaviour—may result in a delayed effect on patient outcome and likely contributes to the observed reduction in overall survival. This interpretation is consistent with the minimal effect of KRT19 on progression‐free survival but its significant association with worse overall survival in patients with high‐grade serous ovarian cancer.

### 4.3 KRT19 in EMT processes

To better understand KRT19‐mediated regulatory processes, we investigated its role in EMP and described the dynamic processes of EMT and MET [28, 37]. The transformation from an epithelial to a mesenchymal cell type is associated with increased stem cell properties, enabling cells to leave the primary tumour [2, 32]. It was observed that EMP is associated with a so‐called “cadherin switch”, in which the expression of epithelial markers decreases, whereas the level of mesenchymal markers increases [32]. Whereas N‐cadherin and vimentin are typical mesenchymal markers, E‐cadherin is a highly expressed epithelial marker and is widely associated with a more favourable outcome in cancer patients across various cancer entities [27]. Nevertheless, studies are controversially discussing the role of EMP markers [11]. In the meantime, it is known that regaining epithelial characteristics is crucial for tumour cells to promote tumour growth at the metastatic site [38]. Therefore, tumour cells exhibit a hybrid epithelial–mesenchymal phenotype. Histopathological evaluation of paired primary and secondary tumours revealed that the majority of metastatic lesions exhibited epithelial morphology and high E‐cadherin expression, which was associated with increased metastatic seeding [39].

To investigate a potential correlation between KRT19 expression and EMP markers, we analysed E‐cadherin, N‐cadherin, and vimentin expression levels in KRT19‐overexpressing HGSOC cell lines compared to control cells. We observed a marked increase in E‐cadherin expression in KRT19‐positive cells, at both the protein and mRNA levels, suggesting that KRT19 may regulate E‐cadherin expression. Previous studies have implicated KRT19 in modulating signalling pathways such as β‐catenin/RAC1 and Notch, which are known to influence EMT and may account for changes in cadherin expression [40]. In contrast, no significant differences were observed in the expression of the mesenchymal marker vimentin at either the transcriptional or translational level, consistent with findings reported by Communal et al. [25].

To explore the seemingly counterintuitive link between KRT19 expression and enhanced migratory behaviour, we performed GSEA comparing KRT19‐overexpressing cells with empty vector controls. This approach aimed to uncover potential interactions between KRT19 and pathways associated with EMP and cell migration.

Our analysis revealed a moderate but consistent enrichment of key signalling cascades known to govern migratory and EMT‐related processes, including the Notch, β‐catenin/Wnt, and RAC1 pathways. The Notch signalling pathway is well established in maintaining cellular plasticity and promoting traits such as motility and invasiveness, particularly in the context of EMT [41]. Likewise, activation of β‐catenin/Wnt signalling has been widely associated with tumour progression and metastasis, largely through its regulation of cell–cell adhesion and cytoskeletal remodelling [42].

While these pathways are classically linked to a mesenchymal phenotype, previous studies have demonstrated that RAC1 activity can promote lamellipodia formation and directional migration even in epithelial cells with intact E‐cadherin junctions, such as MDCK cells [43]. This suggests that enhanced migratory capacity can occur without a complete loss of epithelial features. In line with this, we propose that in cells with a highly mesenchymal background, the partial reacquisition of epithelial traits, such as increased KRT19 and E‐cadherin expression, may confer a functional advantage for migration and metastasis.

Given that both OV‐MZ‐6 and Kuramochi cell lines were derived from ascitic fluid and thus display mesenchymal traits, these results support the hypothesis that increased KRT19 expression may contribute to the aggressiveness of HGSOC cells by promoting E‐cadherin upregulation in ATCs. To further validate these findings, we isolated tumour cells from the ascitic fluid of HGSOC patients and stratified them into KRT19‐high and KRT19‐low groups. High KRT19 expression was consistently associated with elevated E‐cadherin levels, while no correlation was found with mesenchymal markers.

These observations reinforce the notion that KRT19 supports the acquisition of epithelial properties in mesenchymal and detached tumour cells, leading to a hybrid E/M phenotype, and may increase tumour aggressiveness. Together with our previous findings showing that KRT19 promotes intercellular adhesion and reattachment to ECM proteins, the present data suggest that KRT19 may contribute to a more aggressive tumour phenotype by upregulating E‐cadherin, a key mediator of adhesion [44]. This finding may explain the increased tumour burden observed *in vivo*. While overall cell proliferation remains unchanged upon KRT19 overexpression, the formation and growth of metastases by ascites tumour cells appear to be enhanced by a more epithelial phenotype, consistent with the existing literature [45, 46].

### 4.4 KRT19 in chemoresistance

KRT19 is found in various malignancies and has been linked to the development of an epithelial phenotype. Accumulating evidence indicates that elevated KRT19 levels can influence resistance to frontline chemotherapy agents such as paclitaxel and carboplatin. The underlying mechanisms fostering the tolerability of chemotherapy comprise (1) cytoskeletal remodelling resulting in a reinforced cytoskeletal framework [47], (2) EMP [48], (3) induction of survival pathways to overcome DNA damage [49], and (4) stem cell‐like features promoting relapse after treatment [50].

The literature reports opposing effects of KRT19 on chemoresistance, depending on the target cell line or cancer type. Concerning sensitivity against doxorubicin treatment in the breast cancer cell lines MCF7 and BT549, Sharma et al. [32] reported opposing results. In MCF‐7 KRT19 knockdown cells, the resistance towards doxorubicin was increased, whereas in KRT19 overexpressing BT549 cells, the sensitivity decreased towards doxorubicin.

Our findings regarding the two HGSOC first‐line chemotherapies, paclitaxel and carboplatin, align with these inconsistent observations. In the OV‐MZ‐6 cell line, we observed a significant decrease in paclitaxel sensitivity due to increased KRT19 levels. However, in the Kuramochi cell line, KRT19 did not appear to affect chemotherapy sensitivity. This discrepancy may be attributed to the already high basal KRT19 expression in Kuramochi cells. Regarding carboplatin, we found no evidence that KRT19 affects chemosensitivity in either cell line; however, we observed that Kuramochi cells were generally more sensitive to treatment than OV‐MZ‐6 cells. The breast cancer gene (BRCA) 1 and 2 mutations present in the Kuramochi cell line but absent in OV‐MZ‐6 may contribute to the generally increased chemosensitivity observed in Kuramochi cells, indicating a potential role of BRCA‐associated DNA repair deficiency in mediating a more efficient treatment response *per se*, without KRT19 involvement [51] (Table S2). When incorporating *in silico* analyses of HGSOC patient data, KRT19 appears to significantly reduce paclitaxel sensitivity. Similar effects can be seen when stratifying the cohort analysed in this study by carboplatin monotherapy and combined treatment with Taxol. High expression of KRT19 remains associated with poor survival only when Taxol treatment is complementing the therapy regimen. This suggests that KRT19 generally influences paclitaxel chemosensitivity but does so inconsistently across cell lines. This discrepancy between the paclitaxel resistance associated with KRT19 in patient data and the weak *in vitro* effect aligns with multiple reports indicating that microenvironmental factors and tumour heterogeneity, present *in vivo* but absent in cell culture, are critical for fully manifesting chemoresistance mechanisms [52].

## 5 Conclusion

In summary, our study highlights the multifaceted role of KRT19 in HGSOC and reveals its unfavourable prognostic impact on this subtype of EOC. Specifically, KRT19 appears to act as a tumour driver by promoting migratory capacity, improving ATC survival through enhanced intercellular adhesion, and facilitating reattachment to ECM proteins—thereby putatively promoting metastatic dissemination. Those KRT19‐driven effects might be linked to strengthening a hybrid epithelial/mesenchymal phenotype. While additional investigations are needed to elucidate KRT19‐induced chemoresistance, our findings suggest that KRT19 may serve as a negative predictor of response to paclitaxel‐based regimens, with no comparable effect observed with carboplatin. Collectively, these results strengthen the rationale for further research into KRT19 as both a potential biomarker and a therapeutic target in HGSOC.

## Conflict of interest

MK reports remuneration by Springer Press, Biermann Press, Celgene, Astra Zeneca, Myriad Genetics, TEVA, Eli Lilly, GSK, consulting for Myriad Genetics, Bavarian KVB, DKMS Life, BLAEK, TEVA, Exeltis, equity ownership in Therawis Diagnostic GmbH, AIM GmbH, funding from Sphingotec, Deutsche Krebshilfe, DFG, Senator Roesner Foundation, Dr. Pommer‐Jung Foundation, Waltraut Bergmann Foundation, Bavarian State Ministry of Economy, BMBF. HB reports grants from the German Research Foundation (DFG) during the study and personal fees from Roche, AstraZeneca, MSD, Pharma&, AbbVie, and GlaxoSmithKline outside the submitted work. The other authors declare no potential conflicts of interest.

## Author contributions

SB, TD and VM conceived and designed the study. SB, IV, AB, JM, VH, KT, MS, SN and OS acquired data. SB, IV, VM, HB, VK, MK and TD drafted and revised the manuscript. VM and MK gave final approval for publishing the manuscript. All authors read and approved the final manuscript.

## Ethics statement

The use of human samples was approved by the Institutional Review Board of the Technical University of Munich (Munich, Germany; approval 392/17S). Animal experiments were approved by the Government of Upper Bavaria (Regierung von Oberbayern) and were in accordance with the institutional guidelines of the Technical University of Munich. The study was performed in accordance with the Declaration of Helsinki.

## Supporting information


**Fig. S1.** KRT19 does not affect cell morphology. (A) Representative images of OV‐MZ‐6 cells with and without KRT19 overexpression. (B) Representative images of Kuramochi cells with and without KRT19 overexpression. Images were acquired using the EVOS 2000 imaging system at 10× magnification. Shown are representative images of regular cell culture dishes, scale bar 50 μm (*n* = 8).

**Fig. S2.** KRT19 expression in wild‐type cells and validation of overexpression at the protein level. Protein extracts were generated from cell lines transfected with the KRT19 expression plasmid (high) or the empty vector control (low). (A) Endogenous KRT19 expression in three different wild‐type cell lines (OV‐MZ‐6, Kuramochi, OVCAR‐3) (*n* = 1). (B) Validation of KRT19 overexpression in OV‐MZ‐6 cells (*n* = 3). (C) Validation of KRT19 overexpression in Kuramochi cells (*n* = 3).

**Fig. S3.** KRT19 affects EMT marker expression at the protein level in OV‐MZ‐6 cells. Protein extracts were generated from cell lines transfected with the KRT19 expression plasmid (high) or the empty vector control (low). (A) Vimentin expression in KRT19‐overexpressing OV‐MZ‐6 cells compared to control cells (*n* = 3). (B) E‐cadherin expression in KRT19‐overexpressing OV‐MZ‐6 cells compared to control cells (*n* = 3).

**Fig. S4.** KRT19 promotes chemoresistance to paclitaxel. (A) OV‐MZ‐6 and Kuramochi cells were treated with different concentrations of paclitaxel to assess treatment response. (B) OV‐MZ‐6 and Kuramochi cells were treated with different concentrations of carboplatin. No KRT19‐dependent differences in treatment response were observed in either cell line. Error bars depict SEM of three independent experiments (*n* = 3). Statistical significance was determined via an unpaired T‐test for each time point separately; *P < 0.05; error bars +/− SEM.

**Fig. S5.** KRT19 supports migration and metastasis. (A) Invasion of OV‐MZ‐6 cells KRT19+ vs vector control into alginate gel containing 10% FCS as a chemoattractant after different time points. Error bars depict SEM of three independent experiments (*n* = 3). Values are relative to empty vector control. (B) Representative pictures of DAPI‐stained nuclei of migrated OV‐MZ‐6 cells.

**Fig. S6.** *In silico* analysis of KRT19 expression in healthy ovaries and benign ovarian lesions. *Z*‐scores of KRT19 expression derived from publicly available transcriptomic datasets. Data were obtained from the NCBI Gene Expression Omnibus (GEO) repository (GEO accession numbers 41 498, 67 224). *Z*‐score normalisation was applied to enable comparison across samples.

**Table S1.** Patient cohort from TUM University Hospital Rechts der Isar.

**Table S2.** Genomic alterations identified via TruRisk Panel NGS analysis, which may be relevant to mechanisms of chemoresistance.

## Data Availability

The data generated in this study are available upon request from the corresponding author. The publicly available data were accessed via the KM plotter portal (https://kmplot.com/analysis/index.php?p=service&cancer=ovar) and Xenabrowser (https://xenabrowser.net/).
