# Supplementary material for: Keratin 19 as a prognostic marker and contributing factor of metastasis and chemoresistance in high‐grade serous ovarian cancer
Source: Mol Oncol. 2026 Feb 19;20(9):2243–61. doi: 10.1002/1878-0261.70227 (PMC13399148; doi:10.1002/1878-0261.70227)
Supplement: Supplementary file 1 [file MOL2-20-2243-s001.pdf]

Table S2: Genomic alterations identified via TruRisk Panel NGS analysis which may be relevant to mechanisms of chemoresistance.

Cell line	Gene	Alternation type	Details[a)]
**OV-MZ-6**	*BRIP1*	copy number variation	exon 2-20: $\log_2$ fold change +1.97
	FANCM	copy number variation	exon 1-23: $\log_2$ fold change +1.41
	PPM1D	copy number variation	exon 2-6: $\log_2$ fold change +1.82
	RAD51C	copy number variation	exon 3-9: $\log_2$ fold change +1.82
	SLX4	copy number variation	exon 2-15: $\log_2$ fold change -2.00
	XRCC2	copy number variation	exon 1-3: $\log_2$ fold change +1.30
	NF1	frameshift mutation	c.5904_8914del, p.(Gln1969Cysfs*4) (VF 45%)
**Kuramochi**	*BRCA1*	copy number variation	exon 1-24: $\log_2$ fold change -1.25
	BRIP1	copy number variation	exon 2-20: $\log_2$ fold change -1.25
	MLH1	copy number variation	exon 2-19: $\log_2$ fold change -1.26
	RAD51D	copy number variation	exon 1-10: $\log_2$ fold change -2.39
	TP53	copy number variation	exon 2-11: $\log_2$ fold change +1.57
	XRCC2	copy number variation	exon 1-3: $\log_2$ fold change +2.00
	BRCA2	nonsense mutation	c.6952C>T, p.(Arg2318*) (VF 65%)
	TP53	missense mutation	c.841G>T, p.(Asp281Tyr) (VF 99%)

[a)] Fold change values were generated by comparing normalised read depths across targeted regions relative to reference data. Negative or positive $\log_2$ fold change values indicate loss and gain, respectively. The variant fraction (VF) shows the amount of mutated reads for the respective position.
