# Supplementary material for: Keratin 19 as a prognostic marker and contributing factor of metastasis and chemoresistance in high‐grade serous ovarian cancer
Source: Mol Oncol. 2026 Feb 19;20(9):2243–61. doi: 10.1002/1878-0261.70227 (PMC13399148; doi:10.1002/1878-0261.70227)
Supplement: Supplementary file 2 [file MOL2-20-2243-s007.pdf]

Table S1 Patient cohort from TUM University Hospital Rechts der Isar
Tissue samples were used for immunohistochemical stainings of high-grade serous ovarian cancer (HGSOC)

Characteristic		
**Median age at diagnosis [years]**		65
≤ 60	69	
> 60	130	
**Median follow-up time [months]**		33
**FIGO stage**		
III	148	
IV	51	
**Postsurgical residual tumor mass [mm]**		
≤ 10	88	
> 10	107	
Missing data	4	
**Nodal status**		
Negative (pN0)	55	
positive (pN1)	117	
Missing data	27	
**Therapy regimen**		
Carboplatin monotherapy	54	
Carboplatin + Taxol	132	
Therapy terminated or refused	13	
