# Supplementary figures and images for: Keratin 19 as a prognostic marker and contributing factor of metastasis and chemoresistance in high‐grade serous ovarian cancer
Source: Mol Oncol. 2026 Feb 19:10.1002/1878-0261.70227. Online ahead of print. doi: 10.1002/1878-0261.70227 (PMC13399148; doi:10.1002/1878-0261.70227)

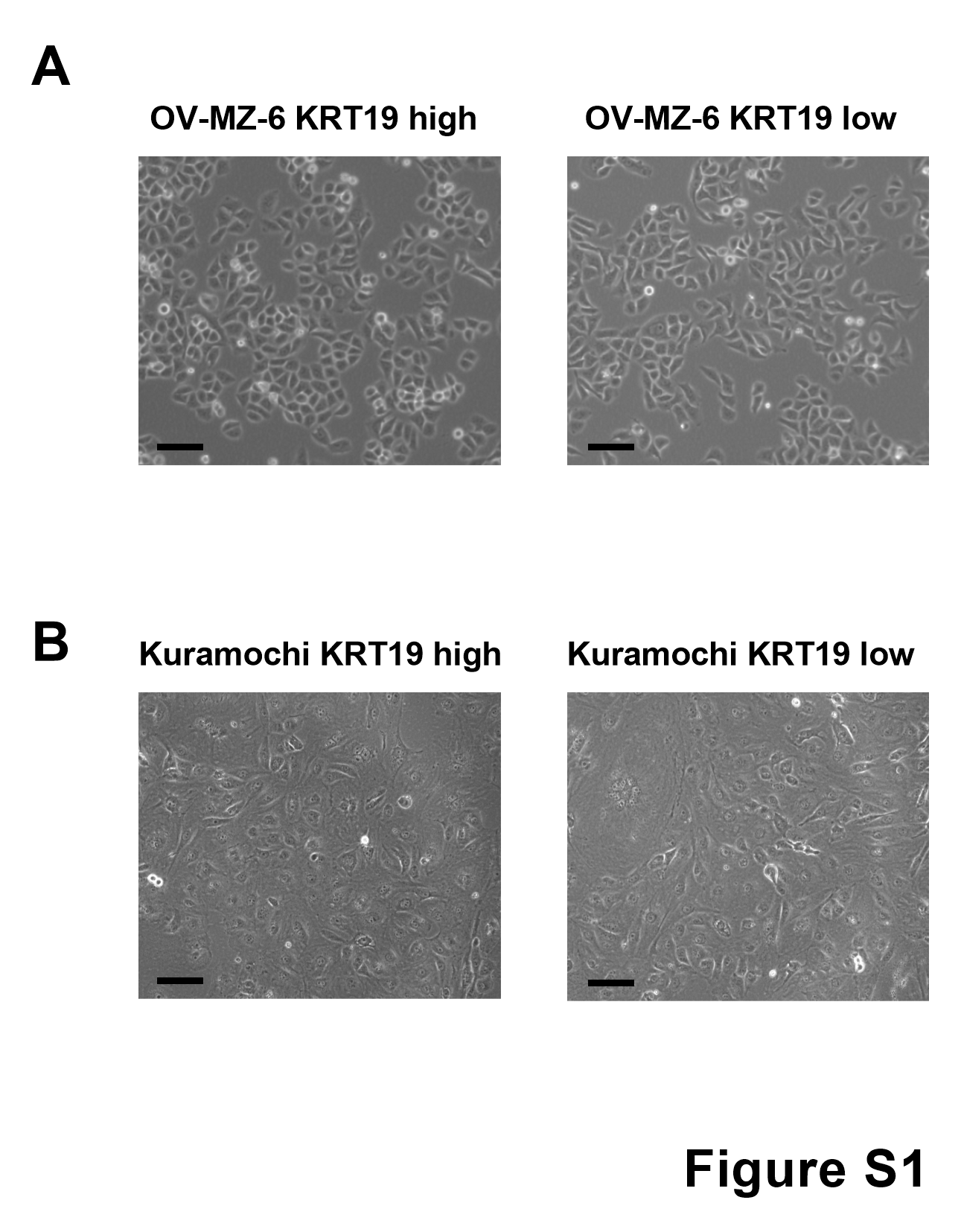

Supplement: Supplementary file 1 — Fig. S1. KRT19 does not affect cell morphology. (A) Representative images of OV‐MZ‐6 cells with and without KRT19 overexpression. (B) Representative images of Kuramochi cells with and without KRT19 overexpression. Images were acquired using the EVOS 2000 imaging system at 10× magnification. Shown are representative images of regular cell culture dishes, scale bar 50 μm (n = 8). [file MOL2-9999-0-s002.tif]

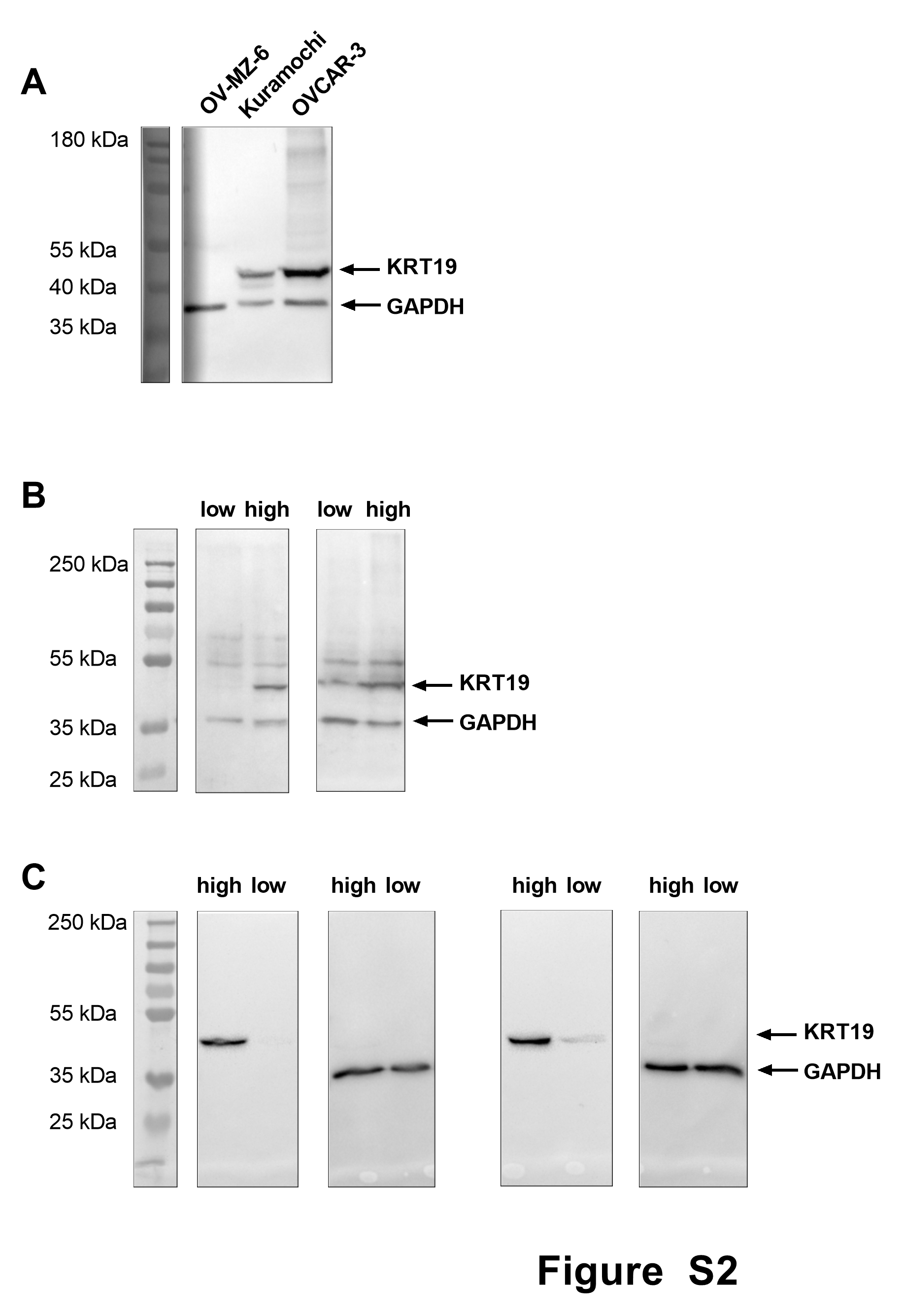

Supplement: Supplementary file 2 — Fig. S2. KRT19 expression in wild‐type cells and validation of overexpression at the protein level. Protein extracts were generated from cell lines transfected with the KRT19 expression plasmid (high) or the empty vector control (low). (A) Endogenous KRT19 expression in three different wild‐type cell lines (OV‐MZ‐6, Kuramochi, OVCAR‐3) (n = 1). (B) Validation of KRT19 overexpression in OV‐MZ‐6 cells (n = 3). (C) Validation of KRT19 overexpression in Kuramochi cells (n = 3). [file MOL2-9999-0-s003.tif]

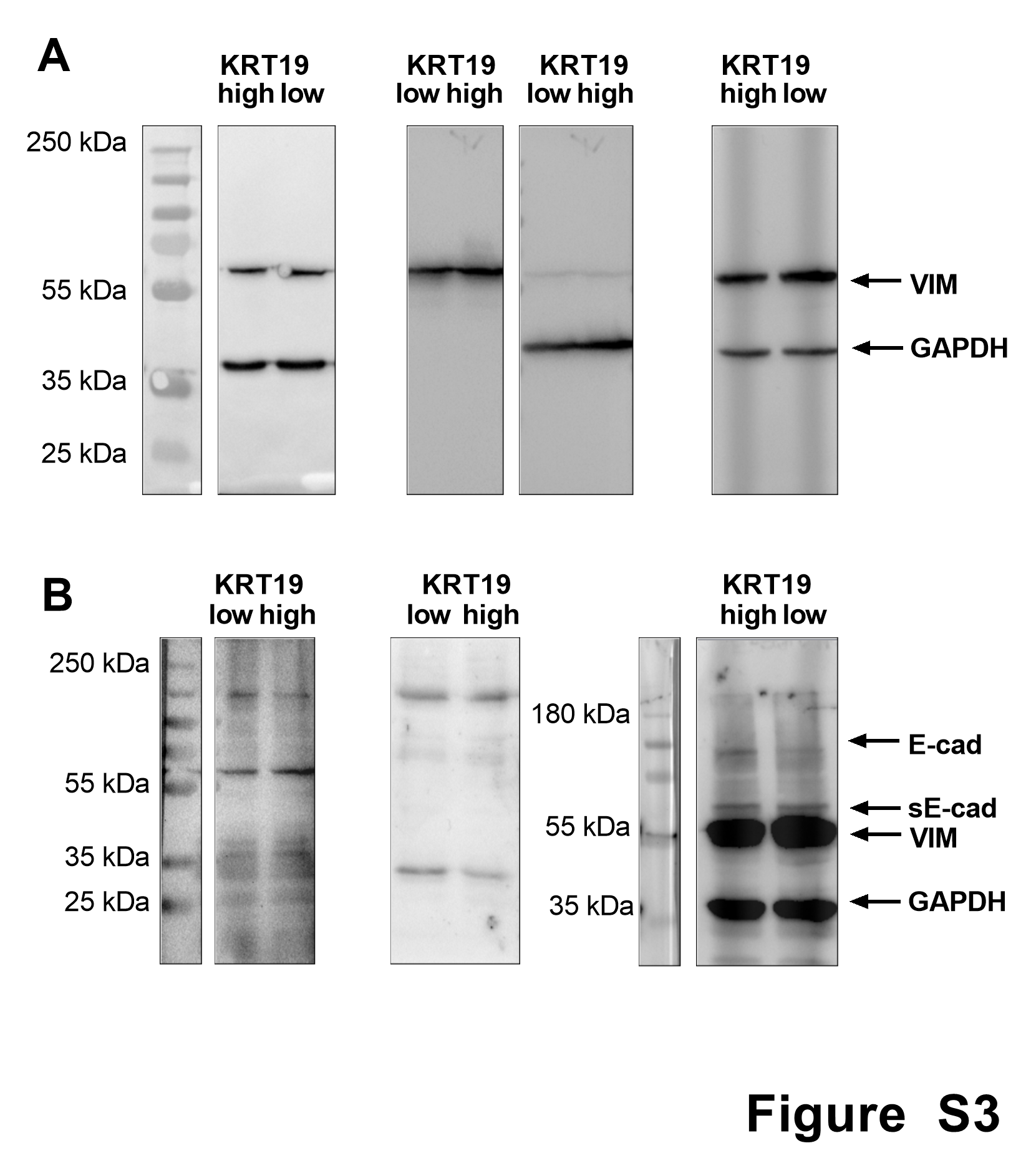

Supplement: Supplementary file 3 — Fig. S3. KRT19 affects EMT marker expression at the protein level in OV‐MZ‐6 cells. Protein extracts were generated from cell lines transfected with the KRT19 expression plasmid (high) or the empty vector control (low). (A) Vimentin expression in KRT19‐overexpressing OV‐MZ‐6 cells compared to control cells (n = 3). (B) E‐cadherin expression in KRT19‐overexpressing OV‐MZ‐6 cells compared to control cells (n = 3). [file MOL2-9999-0-s005.tif]

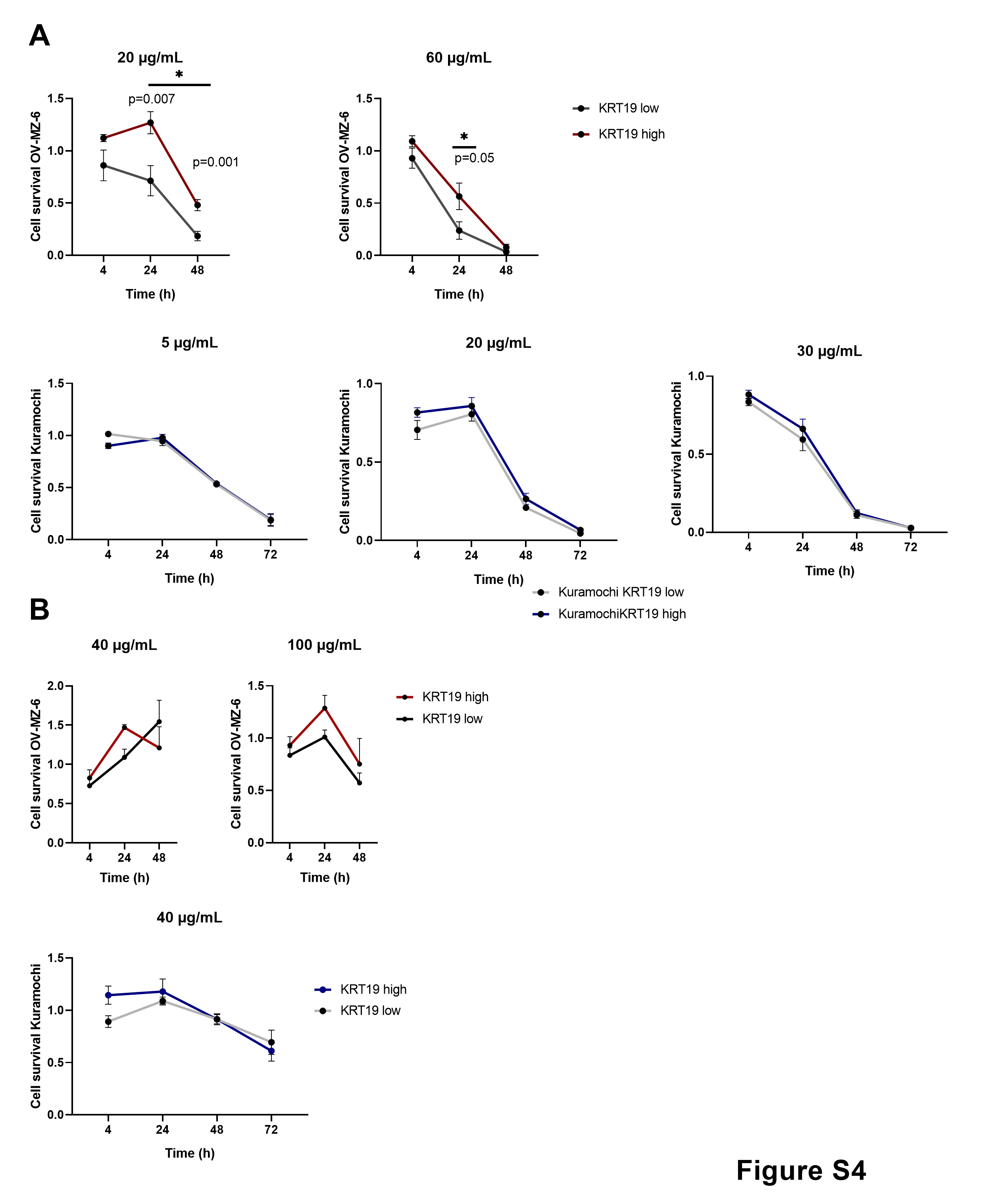

Supplement: Supplementary file 4 — Fig. S4. KRT19 promotes chemoresistance to paclitaxel. (A) OV‐MZ‐6 and Kuramochi cells were treated with different concentrations of paclitaxel to assess treatment response. (B) OV‐MZ‐6 and Kuramochi cells were treated with different concentrations of carboplatin. No KRT19‐dependent differences in treatment response were observed in either cell line. Error bars depict SEM of three independent experiments (n = 3). Statistical significance was determined via an unpaired T‐test for each time point separately; *P < 0.05; error bars +/− SEM. [file MOL2-9999-0-s008.tif]

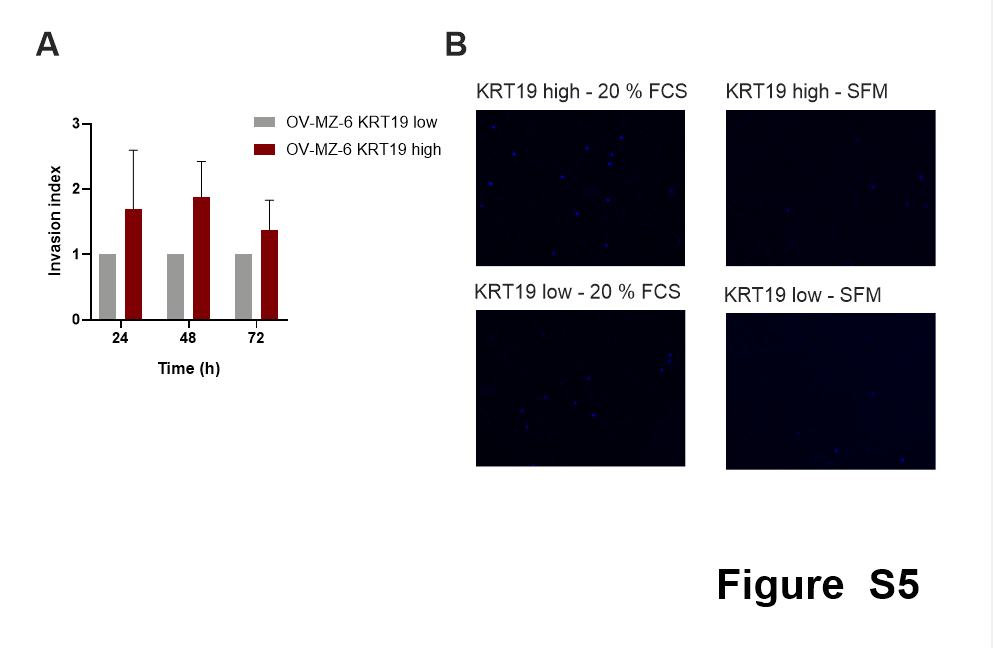

Supplement: Supplementary file 5 — Fig. S5. KRT19 supports migration and metastasis. (A) Invasion of OV‐MZ‐6 cells KRT19+ vs vector control into alginate gel containing 10% FCS as a chemoattractant after different time points. Error bars depict SEM of three independent experiments (n = 3). Values are relative to empty vector control. (B) Representative pictures of DAPI‐stained nuclei of migrated OV‐MZ‐6 cells. [file MOL2-9999-0-s001.tif]

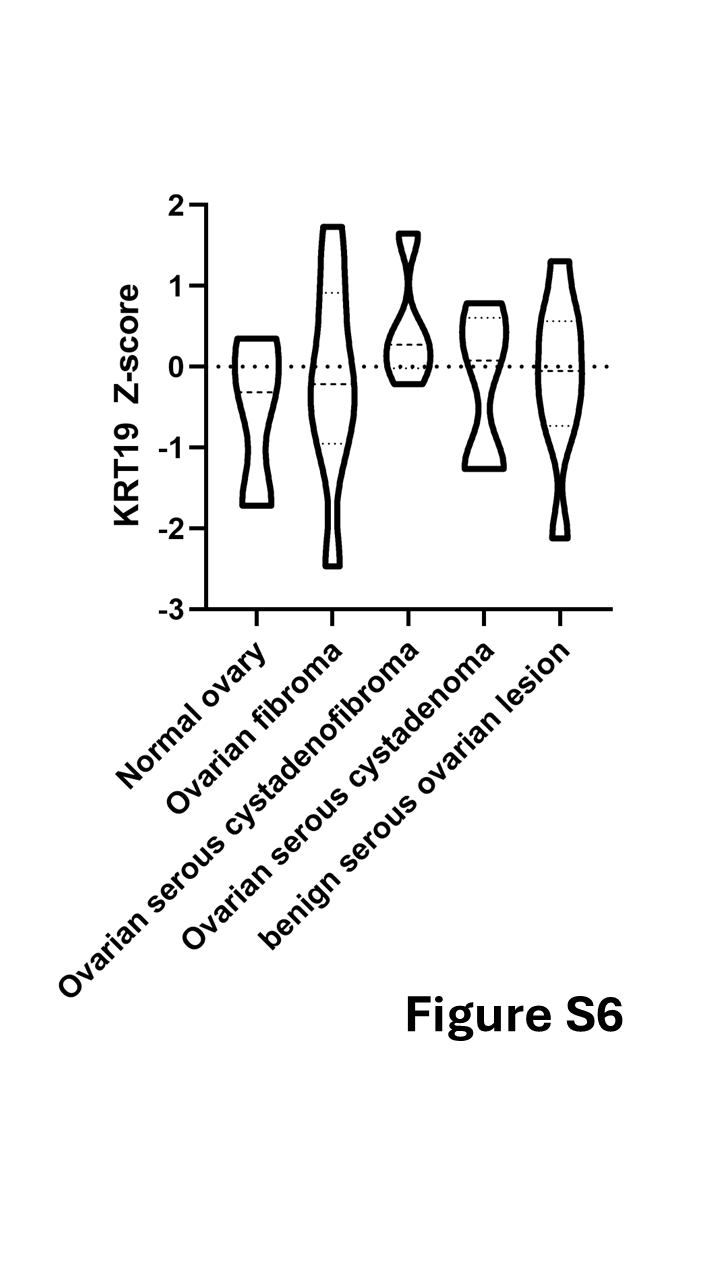

Supplement: Supplementary file 6 — Fig. S6. In silico analysis of KRT19 expression in healthy ovaries and benign ovarian lesions. Z‐scores of KRT19 expression derived from publicly available transcriptomic datasets. Data were obtained from the NCBI Gene Expression Omnibus (GEO) repository (GEO accession numbers 41 498, 67 224). Z‐score normalisation was applied to enable comparison across samples. [file MOL2-9999-0-s006.tif]
